# Identification of differentially expressed plasma small extracellular vesicles miRNAs as biomarkers for type 2 diabetes

**DOI:** 10.3389/fendo.2026.1887230

**Published:** 2026-09-09

**Authors:** Yong Ling Sou, Shamsul Mohd Zain, William M. Chilian, Tilakavati Karupaiah, Faiz Daud, Zulfitri Azuan Mat Daud, Fazliana Mansor, Mohd Fairulnizal Md Noh, Wickneswari Ratnam, Jun-Hao Lim, Cordelia Kheng-May Lim, Catriona Kar-Yuen Ong, Yu-Qiong Chin, Hui-Ching Low, Nur Afrina Muhamad Hendri, Tengku Rogayah Tengku Abd Rashid, Yuh-Fen Pung

**Affiliations:** 1 Division of Biomedical Science, Faculty of Science and Engineering, University of Nottingham Malaysia, Semenyih, Selangor, Malaysia; 2 Department of Pharmacology, Faculty of Medicine, University of Malaya, Kuala Lumpur, Malaysia; 3 Department of Integrative Medical Sciences, Northeast Ohio Medical University, Rootstown Township, OH, United States; 4 School of Biosciences, Faculty of Health and Medical Sciences, Taylor’s University, Subang Jaya, Selangor, Malaysia; 5 Department of Public Health Medicine, Faculty of Medicine, Universiti Kebangsaan Malaysia, Kuala Lumpur, Malaysia; 6 Department of Dietetics, Faculty of Medicine and Health Sciences, Universiti Putra Malaysia, Serdang, Selangor, Malaysia; 7 Nutrition, Metabolism and Cardiovascular Research Centre, Institute For Medical Research, National Institute of Health, Ministry Of Health Malaysia, Shah Alam, Selangor, Malaysia; 8 Department of Biological Sciences and Biotechnology, Faculty of Science and Technology, Universiti Kebangsaan Malaysia, Bangi, Selangor, Malaysia; 9 School of Food Studies and Gastronomy, Faculty of Social Sciences and Leisure Management, Taylor’s University, Subang Jaya, Selangor, Malaysia; 10 Department of Electron Microscopy, Special Resource Centre, Institute of Medical Research, National Institute of Health, Ministry of Health Malaysia, Shah Alam, Selangor, Malaysia

**Keywords:** biomarkers, exosomes, hyperglycemia, insulin resistance, precision medicine

## Abstract

**Background:**

The prevalence of Type 2 Diabetes (T2D) has increased over the years. However, the current diagnostic markers such as fasting blood glucose and HbA1c come with certain limitations. Hence, there is a need for a more robust minimally invasive biomarker. This study aimed to identify sets of dysregulated miRNAs in plasma small extracellular vesicles (sEVs) as biomarkers for T2D diagnosis.

**Methods:**

This study was part of the Rice Intervention for Chronic Health (RICH) trial. Plasma sEVs were isolated from T2D and control subjects and characterized. The sEVs-miRNAs expressions were compared using next generation sequencing and validated using digital PCR. Bioinformatic analyses were performed to study the roles of dysregulated miRNAs. Plasma proteins were profiled to study the systemically dysregulated T2D pathways.

**Results:**

The levels of miR-142-3p and miR-454-3p were increased in plasma sEVs of T2D subjects. Among a subgroup of T2D subjects in whom glycemic control improved (n = 13), these two upregulated miRNAs also decreased. The combination of sEVs-miR-142-3p, waist circumference, and BMI demonstrated high diagnostic values (AUC-ROC=0.985, *p* < 0.001) to distinguish between T2D and control subjects. The integrated results from miRNA bioinformatics analysis and plasma proteomics profiling suggested association between the predicted targets of miRNA and systemic dysregulated pathways in T2D.

**Conclusion:**

This study identified the potential of sEV-miR-142-3p as biomarkers for T2D diagnosis when used in combination with waist circumference and BMI. This study provided preliminary evidence of sEVs-miRNAs in future clinical use for T2D management.

## 1 Introduction

Type 2 diabetes (T2D) is a complex metabolic disease that has posed a significant health burden on the global healthcare system over the past decades (1). The 2025 Diabetes Atlas of the International Diabetes Federation (IDF) reported that nearly 580 million people are currently diagnosed with T2D (2). There is a congruence of T2D with rising obesity globally according to the Global Burden of Disease, and this is in tandem with the rapid globalization and nutrition transitioning to Westernized dietary patterns (3, 4). In Malaysia, which is one of the ‘fattest’ countries in Southeast Asia, 1 in 6 (15.6%) adults are living with T2D (5). The majority of the patients in primary care settings had poor glycemic control, and over 68% of them had glycated hemoglobin (HbA1c) values of >6.5% despite being put under diet control, oral hypoglycemic medications, or insulin treatment (6). At the same time, 2 in 5 adults in Malaysia remain undiagnosed (5). These alarming numbers suggest the need for more effective strategies in T2D prevention, diagnosis, monitoring, and treatment.

T2D is mainly attributed to the developmental stages of insulin resistance, which eventually lead to dysfunction of the pancreatic β cells. Currently in Malaysia, T2D is diagnosed when an individual has a fasting plasma glucose (FPG) value ≥7.0mmol/L or HbA1c value ≥6.3% (7). Despite both FPG and HbA1c being widely used in clinical settings, they have their own limitations. Both measurements only reflect the glucose level of an individual but are unable to capture the underlying molecular changes that occur during T2D development. Furthermore, FPG requires at least 8 hours of fasting of a subject to be reliable for clinical interpretation. It is also highly sensitive to pre-analytical handling and thus the blood samples must be analyzed within 30 min to ensure accurate results (8). Evidently, FPG readings also tend to underdiagnose T2D in Southeast Asian countries like Malaysia and Thailand (9, 10). On the other hand, the HbA1c readings are influenced by factors such as red blood cell turnover and disease status as it measures the amount of blood glucose attached to hemoglobin (11). Moreover, the measurements of HbA1c can be inconsistent especially across different ethnic groups (12, 13). For example, Malaysia accepts a lower cut-off value of 6.3%, while New Zealand uses a higher cut-off value of 6.7% (14). Another study proposed that a lower HbA1c threshold value was more appropriate to diagnose T2D in the African American population (15). Thus, HbA1c is always recommended to be paired with FPG for a more reliable diagnosis of T2D (12, 13). As such, much research in recent years is directed toward exploring new diagnostic markers for T2D, especially one that may support a personalized medicine treatment approach. Among many candidates, microRNAs (miRNAs) particularly have drawn much attention (16).

MiRNAs are small non-coding RNAs with a length between 18 and 22 nucleotides that target and regulate messenger RNAs (mRNAs) at the post-transcriptional levels (17). Once bound to a target mRNA, miRNA functions to silence or induce degradation of their mRNA target. They exist in nearly all types of biofluid, either found free-floating, or encapsulated within the small extracellular vesicles (sEVs) (18). Unlike free-floating miRNAs, sEVs protect miRNAs from being degraded by circulating ribonucleases, thus making them a more ideal biomarker candidate (19). Additionally, the loading of miRNAs into sEVs and how they are produced by specific cell types are also governed by a series of unique molecular mechanisms. Hence, the unique signatures of miRNAs within sEVs could reflect the physiological conditions of an individual’s health condition.

Over the past decades, numerous studies have demonstrated how sEVs-miRNAs were incorporated into different diagnostic models to enhance disease diagnosis. For example, serum sEVs-miR-409 was used in combination with a CA19–9 and a few other biomarkers (cell-free DNA concentration, EV-CK18 mRNA, and CD63 mRNA) to distinguish between pancreatic adenocarcinoma and non-cancer controls (AUC = 0.95) (20). Furthermore, the combination of four sEVs-miRNAs (miR-184, miR-1-3p, miR-432-5p, and miR-1246) and body mass index (BMI) was identified as potential biomarkers for newly diagnosed essential hypertension (AUC = 0.966) (21). Therefore, there is also great potential for sEVs-miRNAs to be incorporated into T2D diagnosis, particularly in the Malaysian population.

By combining miRNA analysis and protein profiling, the objectives of this study were to identify sets of dysregulated plasma sEVs-miRNAs in T2D patients as compared to controls, as well as to elucidate the mechanisms of how sEVs-miRNAs are involved in pathways associated with T2D. Moreover, since rice is a staple food among Malaysians, and a contributing factor to the high prevalence of T2D, this study used a locally bred red rice to assess whether sEV-derived miRNA expression changes alongside improvements in glycemic control (22). The locally bred rice, UKMRC9, was previously reported to show a low glycemic index and reduced postprandial insulin response compared with other commercial rice varieties in the Malaysian market (23). Ultimately, this study will shed light on the potential roles of sEVs-miRNAs in T2D diagnosis and monitoring.

## 2 Methods

### 2.1 Ethics approval, subject recruitment and sample collection

The ethics of Rice Intervention for Chronic Health (RICH) study was registered with the National Medical Research Register (NMRR-20-2183-56420) and approved by the Malaysia Medical Research Committee of the Ministry of Health (KKM/NIHSEC/P20-2117), Universiti Putra Malaysia (UPM/TNCPI/RMC/JKEUPM/1.4.18.2), and Universiti Kebangsaan Malaysia (UKM/PPI/111/8/JEP-2020-712). Written consent was obtained from all subjects prior to the study, and all recruitment procedures were conducted in accordance with the Declaration of Helsinki.

The RICH study was a double-blind, randomized controlled trial in Malaysia. The inclusion criteria for control subjects (non-T2D control) were as follows: individuals with HbA1c values <6.3% and no prior blood glucose-lowering treatment; free of other metabolic diseases such as kidney failure, liver and pancreatic diseases, and all types of cancer; not pregnant or undergoing lactation. The inclusion criteria for T2D subjects were as follows: patients clinically diagnosed with T2D by clinicians with HbA1c values between 6.3% and 10%; currently not under insulin treatment, free of kidney failure, liver and pancreatic diseases, all types of cancer, and T2D complications, not pregnant or undergoing lactation. These eligibility criteria were prespecified as part of the trial registration at ClinicalTrials.gov (ID: NCT06560541). Subjects recruited into the RICH study were randomly allocated into two intervention arms for 24 weeks to study the effects of substituting white rice with low-GI red rice on the anthropometry measurements, blood biochemical analysis and small EV miRNAomes. sEVs were isolated from plasma of T2D subjects at baseline and at 24 weeks, and their miRNA content was profiled using next generation miRNA sequencing and confirmed by digital PCR (dPCR). As glycemic parameters are key indicators of T2D condition, changes in sEVs-miRNA expression were evaluated together with the changes in glycemic parameters to assess the potential of sEVs-miRNAs as T2D biomarkers. In this study, only data collected at 24 weeks post-intervention was presented, as no statistically significant differences were observed in clinical parameters at the 12-week time point.

The propensity scores matching method was adapted from *Danielson* et al. (24) to select samples for next generation miRNA sequencing, where logistic regression was first performed to generate propensity scores for each subject from the T2D and control groups, then subjects with the closest propensity scores from each group were considered as the best-match pair (24). The propensity scores were calculated and matched based on gender, ethnicity, and BMI. Age was not included as a matching variable as T2D subjects in the RICH study were significantly older than the controls. Plasma samples for this study were obtained from a sub-cohort of the RICH study. From each subject, 5 mL of whole blood was collected in an EDTA tube and centrifuged at 1,500 x g for 10 min to separate the plasma layer, which was subsequently aliquoted and stored immediately at -80 °C until further use.

### 2.2 Sample size calculation

The sample size estimation for this study was computed using the G*Power 3.1.9 software. Two-tailed t-test was conducted followed by *a priori* power analysis with the following assumptions: effect size= 1.89, α= 0.05, power= 0.95. The effect size was calculated based on the outcomes from previous studies related to sEVs-miRNAs and T2D (25, 26). The minimum number of samples per sample group was n=9, bringing the total up to a minimum of n=27 subjects in this study. Accounting for a potential 30% drop-out rate, and given that samples might not meet quality criteria due to sample lysis, at least n=36 subjects were required. By analyzing a total of n=63 subjects, the study achieved sufficient statistical power.

### 2.3 Isolation of small extracellular vesicles

For each subject, 1.3 mL of aliquoted plasma was used as starting material to isolate sEVs using the miRCURY serum/plasma isolation kit (Cat # 76603, Qiagen, Germany) with minor modifications. sEVs isolated were resuspended in 1x phosphate buffered saline (PBS), then followed by an ultracentrifugation step at 110,000 x g for 70 min at 4 °C to purify the EV isolates (27).

### 2.4 Characterization of isolated small extracellular vesicles

In accordance with the Minimal Information for Studies of Extracellular Vesicles (MISEV) guidelines, the isolated vesicles were characterized using the following methods: transmission electron microscopy (TEM) to observe the ultrastructure; nanoparticle tracking analysis (NTA) to estimate the size and particle concentrations, and western blot to identify different EV markers (28).

#### 2.4.1 Transmission electron microscopy

Isolated sEVs from each sample were first resuspended in a fixative containing 2.5% glutaraldehyde in 1.0 N of phosphate buffer solution (PBS) and dropped onto a copper mesh grid of the electron microscope. The sample was set to dry for 5 min at room temperature before staining with 1% of ammonium molybdate for 10 sec. The excess staining solution was wiped away using filter paper. Finally, the sample was viewed using a Tecnai™ G2 Spirit transmission electron microscope operating at 100 kV (FEI, Hillsboro, OR).

#### 2.4.2 Nanoparticle tracking analysis

The particle concentration and size distribution of isolated sEVs were measured by NTA on the NanoSight NS300 and analyzed with the NTA 3.4 software (Malvern Panalytical, UK). Samples were diluted using deionized water and measured at 5 x 60 sec, a constant temperature of 27 °C and viscosity of 0.849-0.850 cP. The detection threshold of the camera was set to 20, camera type to scMOS, laser type to Blue488, camera level to 16 (NTA 3.0 levels), slider shutter to 1300, slider gain to 295, flow per second to 25.0, and number of frames to 1498.

#### 2.4.3 Western blot analysis

An equal volume of 7.5 μL protein samples diluted in 6x Laemmli buffer was separated by a 12% SDS-PAGE gel under reducing conditions at 80 V and transferred onto polyvinylidene fluoride (PVDF) membranes. The PVDF membranes were blocked in 3% milk or 5% bovine serum albumin (BSA) in 0.1% Tris-buffered saline with Tween 20 (TBST), depending on the primary antibodies used in the subsequent step, for 45 min on a shaker incubator at room temperature. Next, the membranes were incubated overnight at 4 °C in primary antibodies against CD9 (Cat# 13174 1:1000, Cell Signaling Technology, Danver, MA), TSG101 (Cat# MA1-23296 1:2000, Thermo Fisher Scientific, Waltham, MA), and Calnexin (Cat# 2679T 1:1000, Cell Signaling Technology, Danvers, MA). CD9 and TSG101 were used as positive markers to indicate the presence of sEVs, and calnexin was used as a negative marker to indicate non-sEV cellular contamination. The membranes were washed in 0.1% TBST and incubated for 1 hr in respective HRP-conjugated anti-rabbit IgG or anti-mouse IgG (Cat # 7074 & Cat # 7076, Cell Signaling Technology, Danvers, MA). Signals were detected using ClarityTM Western ECL kit (Cat # 1715061, Bio-Rad, Hercules, CA). Finally, the images were captured by the ChemiDoc XRS system (Bio-Rad, Hercules, CA) and analyzed with Image Lab software (Bio-Rad, Hercules, CA).

### 2.5 Total RNA extraction and small-RNA library construction

Total RNA was extracted from sEVs using the miRNeasy Micro kit (Cat # 217084, Qiagen, Germany) according to the manufacturer’s protocol. Next, miRNA libraries were constructed using the QIAseq^®^ miRNA UDI library kit (Cat # 331502, Qiagen, Germany) following the manufacturer’s instructions. Quality check (QC) of constructed libraries was performed using the Qubit 3.0 fluorometer (Thermo Fisher Scientific, Waltham, MA) and Lab Chip GX TouchTM (PerkinElmer, Waltham, MA). Library products that passed quality assessment were normalized and pooled before loading into the Novaseq 6000 platform for sequencing (Illumina, San Diego, CA). The sequencing was run on 75 bp single-end with 10 million read depth. Demultiplexing of samples was performed using Illumina DRAGEN Bio-IT Platform (v3.9).

### 2.6 MiRNA profiling with next generation small-RNA sequencing

After demultiplexing, all single-end raw data in FASTQ format was subjected to another round of QC using FastQC software to obtain basic statistics of sequencing quality. The analysis of unique molecular identifiers (UMI) in miRNA sequences, trimming of 3’/5’ adapters, and read length filtering were done using the Qiagen CLC Genomics Workbench software v23.0.5 (Qiagen, Germany). The retained reads from previous steps were mapped against the human reference genome (GRCh38) and miRbase (version 22) for annotation with up to two mismatches. The edgeR package was used to calculate the log expression of each miRNA and to compare the differential miRNA profiles between different groups. *P*-values were adjusted for multiple testing using the Benjamini-Hochberg procedures in edgeR to control false discovery rate (FDR). The cut-off points for differential expression between groups were set to |log_2_fold change|>1, FDR <0.05.

### 2.7 MiRNA quantification with digital PCR

Digital PCR (dPCR) was performed using samples from both the NGS discovery cohort and an independent validation cohort. The extracted total RNA was reverse transcribed into cDNA using the miRCURY LNA RT kit (Cat # 339340, Qiagen, Germany) following the manufacturer’s instructions. Next, diluted cDNA was combined with nuclease-free water, QIAcuity 3x EG PCR Mastermix (Cat # 250111, Qiagen, Germany), and target primer assays (Cat # 339306, Qiagen, Germany) for each dPCR reaction. Samples were loaded into the QIAcuity™ 8K 96-well Nanoplate (Cat # 250031, Qiagen, Germany) and sealed according to the manufacturer’s instructions. Each reaction was performed in duplicate with a non-template control in accordance with the latest MIQE dPCR guidelines (29). The cycling conditions were set as following the manufacturer’s protocol: initial heat activation at 95 °C for 2 min, denaturation for 15 sec at 95 °C and annealing/extension for 1 min at 60 °C, followed by final cooling down for 5 min at 40 °C. For imaging, the channel was set to green, with an exposure duration of 200 ms and gain value of 6. All expressions were normalized to miR-191-5p, a reference miRNA selected based on stability analysis (**Supplementary Table 1**) (30, 31).

### 2.8 Bioinformatics analysis

The mRNA targets of differentially expressed miRNAs were predicted using TargetScan, MIRTarBase, and miRDB (32–34). Then, a protein-protein interaction (PPI) network was constructed using the STRING database (35). The top 20 HubGenes were identified using Cytoscape v3.10.2 with the CytoHubba plug-in (36, 37). Subsequently, Gene Ontology (GO) enrichment and Kyoto Encyclopedia of Genes and Genomes (KEGG) pathway analysis were performed with the identified HubGenes using the clusterProfiler package in R (38).

### 2.9 Proteomics profiling

The total plasma proteins were measured on the SomaScan^®^ 11K Assay v5.0 (SomaLogic, Boulder, CO). The protein expression results were exported as relative fluorescence units (RFU). The differential expression analysis was performed in R using the limma and dplyr packages. The Gene Set Enrichment Analysis (GSEA) was conducted to elucidate the systemic dysregulated pathways associated with T2D. The analysis was performed using the Hallmark gene sets from the Molecular Signatures Database (MSigDB) on GSEA desktop software v4.4.4.0. Leading-edge analysis was conducted to identify the subsets of genes that were most responsible for hallmark enrichment.

### 2.10 Statistical analysis

Statistical analysis was performed with SPSS version 26.0 (IBM Corp., Armonk, NY) and GraphPad Prism 10 (GraphPad Software, San Diego, CA). The Shapiro-Wilk test was applied to evaluate the normality of data. A Chi-square test was used to compare the differences between two categorical variables. For normally distributed data, a paired t-test or independent t-test was used to compare the difference between two groups. For non-normally distributed data, Mann-Whitney U-test or Wilcoxon signed-rank was used to compare the difference between two groups. Spearman’s rank correlation analysis was used to analyze the correlation between miRNAs and other indicators, depending on the normality of the data. Sex-stratified analysis of independent t-test followed by univariate ANOVA analysis was performed to evaluate the sex-specific effects. Binary logistic regression was used to assess the association between miRNAs and other T2D metabolic parameters. The multivariate logistic regression models were constructed based on forward likelihood ratio calculations. ROC analysis was used to evaluate the sensitivity and specificity of diagnostic indicators. Internal validation was performed in R version 4.6.1 (R Core Team, Vienna, Austria) using bootstrap optimism correction with 1,000 iterations following the multiple imputation method. All normally distributed data were expressed as mean ± standard deviation, while non-normally distributed data were expressed as median (interquartile range), unless otherwise stated. *p* < 0.05 was the cut-off point for statistical significance.

## 3 Results

### 3.1 Subject characteristics

A total of 63 RICH subjects were analyzed in this study. The demographic and clinical characteristics of these 25 controls (non-T2D) and 38 T2D subjects were reported in **Table 1**. The mean age of subjects from control and T2D groups was 35.3 ± 9.0 and 48.6 ± 7.3 years, respectively. Notably, the T2D group was found to have significantly higher body mass index (BMI), waist circumference measure, FPG, HbA1c, triglycerides, systolic blood pressure (SBP), and diastolic blood pressure (DBP) compared to the control group. On the other hand, HDL-C and LDL-C were significantly lower in the T2D group as compared to the control group. Significant reductions in FPG and HbA1c were observed at 24-week post-intervention for T2D subjects that consumed low-GI red rice (**Supplementary Table 2**).

**Table 1:** Subject demographic and clinical characteristics (n=63).

Variable	Control	T2D	*p* value
Age (year)	35.3 ± 9.0	48.6 ± 7.3	<0.001^a*^
Sex			0.793^b^
Male, n (%)	11 (44)	18 (47)	
Female, n (%)	14 (56)	20 (53)	
Ethnicity			0.127^b^
Malay, n (%)	10 (40)	24 (64)	
Chinese, n (%)	10 (40)	7 (18)	
Indian, n (%)	5 (20)	7 (18)	
T2D duration (month)		82.6 ± 66.8	
BMI (kg/m²)	22.9 ± 2.8	31.0 ± 5.3	<0.001^a***^
Waist circumference (cm)	74.7 ± 9.4	95.1 ± 12.0	<0.001^a***^
FPG (mmol/L)	4.76 ± 0.3	8.5 ± 2.3	<0.001^a***^
HbA1c (%)	5.3 ± 0.4	8.1 ± 1.0	<0.001^a***^
Total cholesterol (mmol/L)	5.5 ± 1.2	4.9 ± 1.1	0.051^a^
HDL-C (mmol/L)	1.7 (1.5-1.8)	1.3 (1.1-1.5)	<0.001^c***^
LDL-C (mmol/L)	3.4 ± 1.1	2.8 ± 1.0	0.026^a*^
Triglycerides (mmol/L)	1.0 (0.7-1.0)	1.8 (1.2-2.1)	<0.001^c***^
SBP (mmHg)	118.3 ± 15.5	139.2 ± 19.4	<0.001^a***^
DBP (mmHg)	78.6 ± 8.9	92.1 ± 14.2	<0.001^a***^
Non-insulin glucose lowering drug(s)			
Metformin, n (%)		9 (23.7)	
Sulfonylureas, n (%)		1 (2.6)	
Metformin + Sulfonylureas, n (%)		17 (44.7)	
Metformin + DPP-4 inhibitor, n (%)		1 (2.6)	
Metformin + DPP-4 inhibitor + SGLT2 inhibitor, n (%)		1 (2.6)	
Anti-hypertensive medication(s)			
Calcium-Channel Blocker, n (%)		10 (27.0)¹	
Angiotensin-Converting Enzyme inhibitor, n (%)		10 (27.0)¹	
Angiotensin Receptor Blocker, n (%)		4 (10.8)¹	
Beta Blocker, n (%)		1 (2.7)¹	

Age, BMI, waist circumference, FPG, HbA1c, triglycerides, SBP, and DBP were significantly higher in T2D group, while HDL-C and LDL-C were significantly higher in control group. The lower HDL-C and LDL-C levels observed in the T2D group may be a result of lipid-lowering medications used, which are commonly prescribed to T2D patients for cardiovascular risk management. Data presented as n (%) for categorical data, mean ± SD for normally distributed data, or median (interquartile range) for non-normally distributed data.

^a^ Independent sample t-test.

^b^ Chi-square test.

^c^ Mann-Whitney U test.

¹Percentages are not mutually exclusive as some subjects received more than one class of antihypertensive medication.

BMI indicates body mass index; DBP, diastolic blood pressure; FPG, fasting plasma glucose; HDL-C, high density lipoprotein; LDL-C, low density lipoprotein; SBP, systolic blood pressure; T2D, Type 2 Diabetes. *p<0.05, ***p<0.001.

### 3.2 No significant differences in morphology, size distribution and particle concentration between sEVs isolated from T2D and control subjects

**Figure 1:**
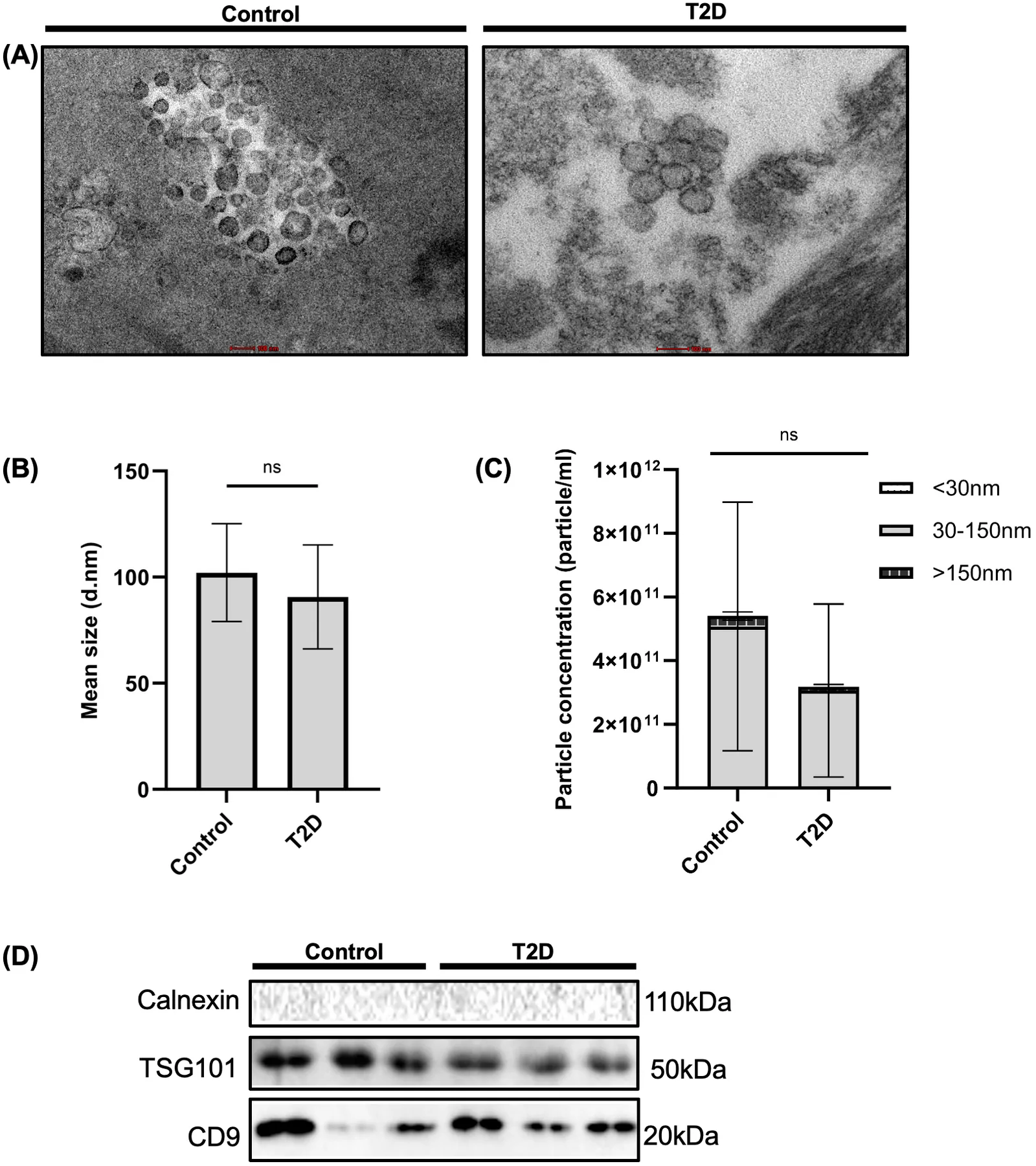
Characterization of isolated sEVs showing no significant differences in terms of morphology, size, particle concentration and protein marker expressions between T2D and control subjects (n=3-6). **(A)** Representative images from TEM showed no major ultrastructure differences between control and T2D group. **(B)** Mean size of isolated sEVs measured using NTA showed no significant differences in particle size between the control and T2D group. **(C)** Particle concentration measured using NTA showed no significant differences in number of particles between the T2D and control. **(D)** Western blot results demonstrated the presence of positive markers (CD9 and TSG101) in sEVs samples isolated from both groups. A positive lysate control for Calnexin was not included. Therefore, the absence of a Calnexin signal should be interpreted with caution and not as definitive evidence of no cellular contamination. Nonetheless, the presence of the two positive sEV markers indicated that sEVs had been successfully isolated for subsequent analysis. TEM indicates Transmission Electron Microscopy; NTA, Nanoparticle Tracking Analysis; T2D, Type 2 Diabetes; sEVs, small extracellular vesicles. Data were presented as mean ± SD.

Isolated sEVs were characterized by the standards of Minimal Information for Studies of Extracellular Vesicles (MISEV) 2023, from their morphology, mean size, particle concentrations, to the presence of EV markers (28). Overall, TEM images demonstrated no major differences in sEV morphology between control and T2D subjects. Both groups displayed a heterogenous population of sEVs with spherical-shaped, ranging between 65–135 nm as measured by TEM and NTA (**Figure 1A**). There were also no significant differences in terms of mean size (*p* = 0.89) and particle numbers (*p* = 0.45) between both groups. The majority of isolated vesicles were found to have sizes between 30–150 nm (**Figures 1B, C**). Furthermore, these vesicles were also enriched in the two commonly reported sEV markers, CD9 and TSG101 (**Figure 1D**).

### 3.3 MiRNA profiling identified 12 significantly dysregulated sEVs-miRNAs in T2D subjects

A total of 985,817,712 reads were obtained from sequencing and passed QC with average Q30(%) of 97.5%. These reads were trimmed and mapped against the human reference genome (GRCh38) and miRbase (v22) to annotate for miRNAs. 179,269,623 (53.47%) and 156,019,602 (51.46%) reads from control and T2D groups, respectively, were annotated as miRNAs.

**Figure 2:**
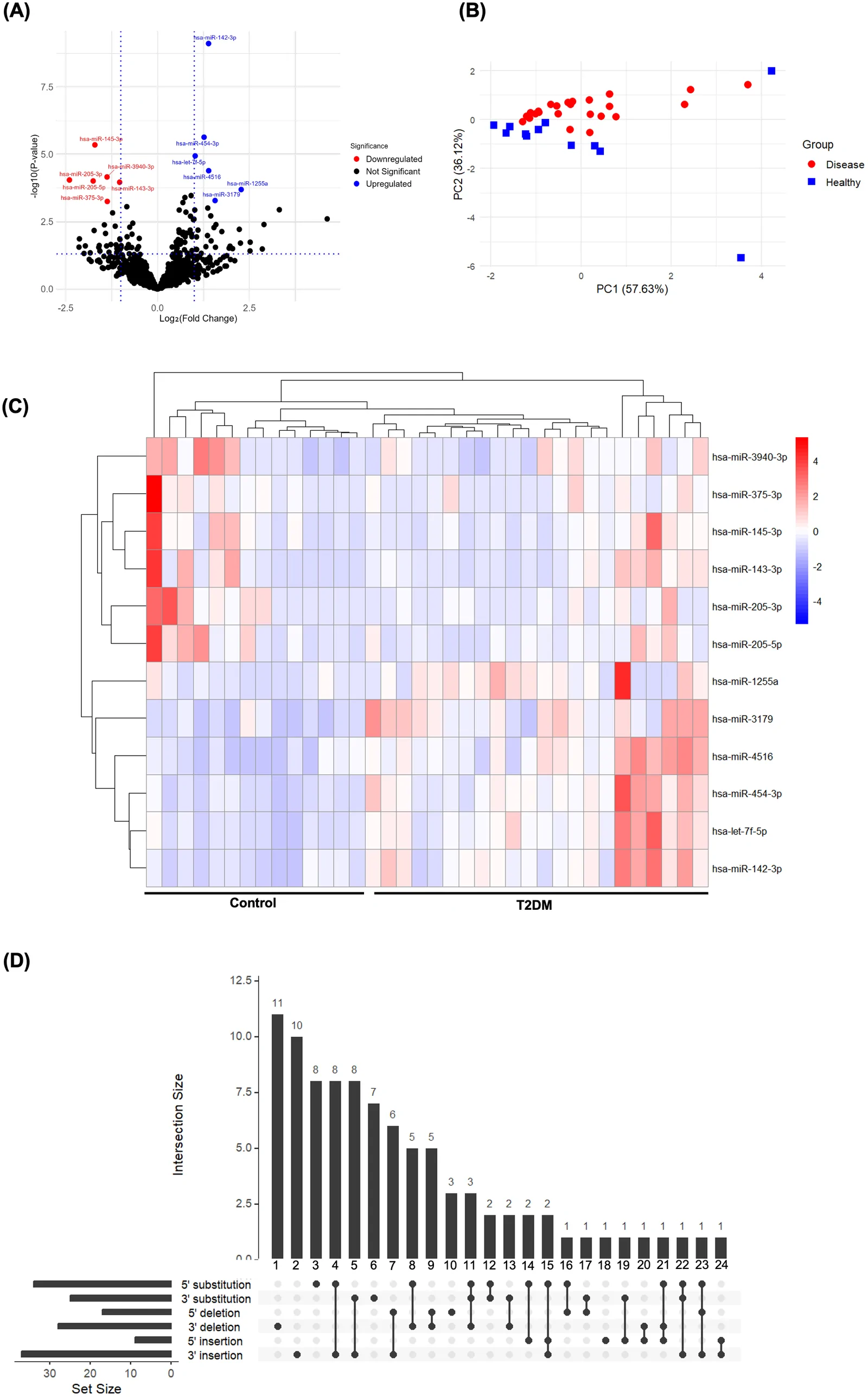
Differential sEVs-miRNAs expression analysis between T2D and control group (n=36). **(A)** Volcano plots showing differentially expressed miRNAs (|log_2_fold change|>1, FDR<0.05) in red and blue. 6 miRNAs were found to be upregulated and downregulated, respectively, in plasma sEVs of T2D as compared to control group. **(B)** Principal component analysis of differentially expressed miRNAs in serum sEVs showed distinct separation between T2D and control group. **(C)** Hierarchical cluster analysis of differentially expressed sEVs miRNAs between T2D and control group. The distinct clusters indicate differential miRNAs expression between T2D and control group. miRNA expressions were normalized using Z-score to construct heatmap. **(D)** Upset plot showing types of differentially expressed miRNAs isoforms (isomiRs) between T2D and control subjects. A total of 122,731 isomiRs were identified, however, only 94 isomiRs (43 upregulated and 51 downregulated) have |log_2_fold change|>1 and FDR<0.05. 24 types of differentially expressed isomiRs detected, of which 3’ deletion was the most frequently modified isoform (11.7%), followed by 3’ insertion (10.6%).

A total of 2632 canonical miRNAs were identified in this study, of which, 110 (66 upregulated and 44 downregulated) were differentially expressed between the T2D and control groups (*p<*0.05). However, further analysis demonstrated that only 12 miRNAs (6 upregulated and 6 downregulated) had |log_2_fold change|>1 and FDR<0.05 (**Figure 2A**, **Table 2**). The principal component analysis (PCA) showed a distinct separation between T2D and control groups (**Figure 2B**). Moreover, hierarchical clustering of differentially expressed sEVs-miRNAs showed distinct clusters between T2D and control groups, further supporting the findings from PCA (**Figure 2C**).

**Table 2:** Differentially expressed sEVs-miRNAs between T2D and control group.

Name	Log_2_fold change	*p* value	FDR value
hsa-miR-142-3p	1.388	<0.001	<0.001
hsa-miR-454-3p	1.266	<0.001	0.001
hsa-let-7f-5p	1.025	<0.001	0.003
hsa-miR-4516	1.391	<0.001	0.008
hsa-miR-1255a	2.277	<0.001	0.020
hsa-miR-3179	1.565	<0.001	0.040
hsa-miR-145-3p	-1.705	<0.001	0.001
hsa-miR-3940-3p	-1.374	<0.001	0.012
hsa-miR-143-3p	-1.032	<0.001	0.012
hsa-miR-205-5p	-1.751	<0.001	0.012
hsa-miR-205-3p	-2.398	<0.001	0.012
hsa-miR-375-3p	-1.367	<0.001	0.040

Among the 2632 miRNAs identified, 12 miRNAs were significantly dysregulated (6 upregulated and 6 downregulated) between T2D and control group. miRNAs were considered as differentially expressed when |log_2_fold change|>1 and FDR<0.05.

Despite the majority of miRNAs being annotated as canonical miRNAs (87.5%), a small percentage (12.5%) of them were found to have modifications at 3’ and/or 5’ ends and hence were classified as isomiRs. A total of 122,731 isomiRs were identified, of which, 6,416 isomiRs were dysregulated (1,600 upregulated and 4,816 downregulated) between T2D and control groups (*p<*0.05). Further analysis demonstrated that only 94 miRNAs (43 upregulated and 51 downregulated) had |log_2_ fold change|>1 and FDR<0.05 (**Table 3**, **Supplementary Table 4**). Twenty-four types of modification were detected among those differentially expressed isomiRs, of which 3’ deletion was the most frequently modified isoform (11.7%), followed by 3’ insertion (10.6%) (**Figure 2D**).

**Table 3:** Top 20 differentially expressed (10 upregulated and 10 downregulated) sEVs isomiRs between T2D and control group.

Name	Log_2_fold change	*p* value	FDR value
hsa-miR-4433b-3p.As.G8C	10.96	<0.0001	<0.0001
hsa-miR-4433b-3p.G8C	9.68	<0.0001	<0.0001
hsa-miR-1-3p.T22G	8.73	<0.0001	<0.0001
hsa-miR-4433b-3p.G8C.AT	7.82	<0.0001	<0.0001
hsa-miR-4433b-3p.As.G8C.T	7.78	<0.0001	<0.0001
hsa-miR-100-5p.TT	7.61	<0.0001	<0.0001
hsa-miR-4433b-3p.G8C.T	7.53	<0.0001	<0.0001
hsa-miR-4433b-3p.As.G8C.t	7.33	<0.0001	<0.0001
hsa-miR-4433b-3p.As.G8T	7.20	<0.0001	<0.0001
hsa-miR-184.t	6.52	<0.0001	<0.0001
hsa-miR-192-5p.AT	-5.03	<0.0001	0.0039
hsa-miR-432-5p.G11A.gg	-4.35	<0.0001	0.0068
hsa-miR-320d.Gs.A.T	-4.11	<0.0001	0.0053
hsa-miR-187-5p.gs.G	-3.45	<0.0001	0.0055
hsa-miR-205-5p.g	-2.22	<0.0001	0.0097
hsa-miR-375-3p.ts.a	-2.13	<0.0001	0.0018
hsa-miR-375-3p.ga	-1.91	<0.0001	0.0056
hsa-miR-125b-5p.ga	-1.71	<0.0001	0.0039
hsa-miR-375-3p.a	-1.70	<0.0001	0.0068
hsa-miR-143-3p.T	-1.60	<0.0001	0.0126

Among the 94 dysregulated (43 upregulated and 51 downregulated) isomiRs in sEVs between two groups, only the 20 most significantly altered isomiRs were presented. The full list of dysregulated sEVs-isomiRs was presented in **Supplementary Table 3**. isomiRs were considered as differentially expressed when |log_2_fold change|>1 and FDR<0.05.

### 3.4 MiR-142-3p and miR-454-3p were upregulated in plasma sEVs of T2D subjects

**Figure 3:**
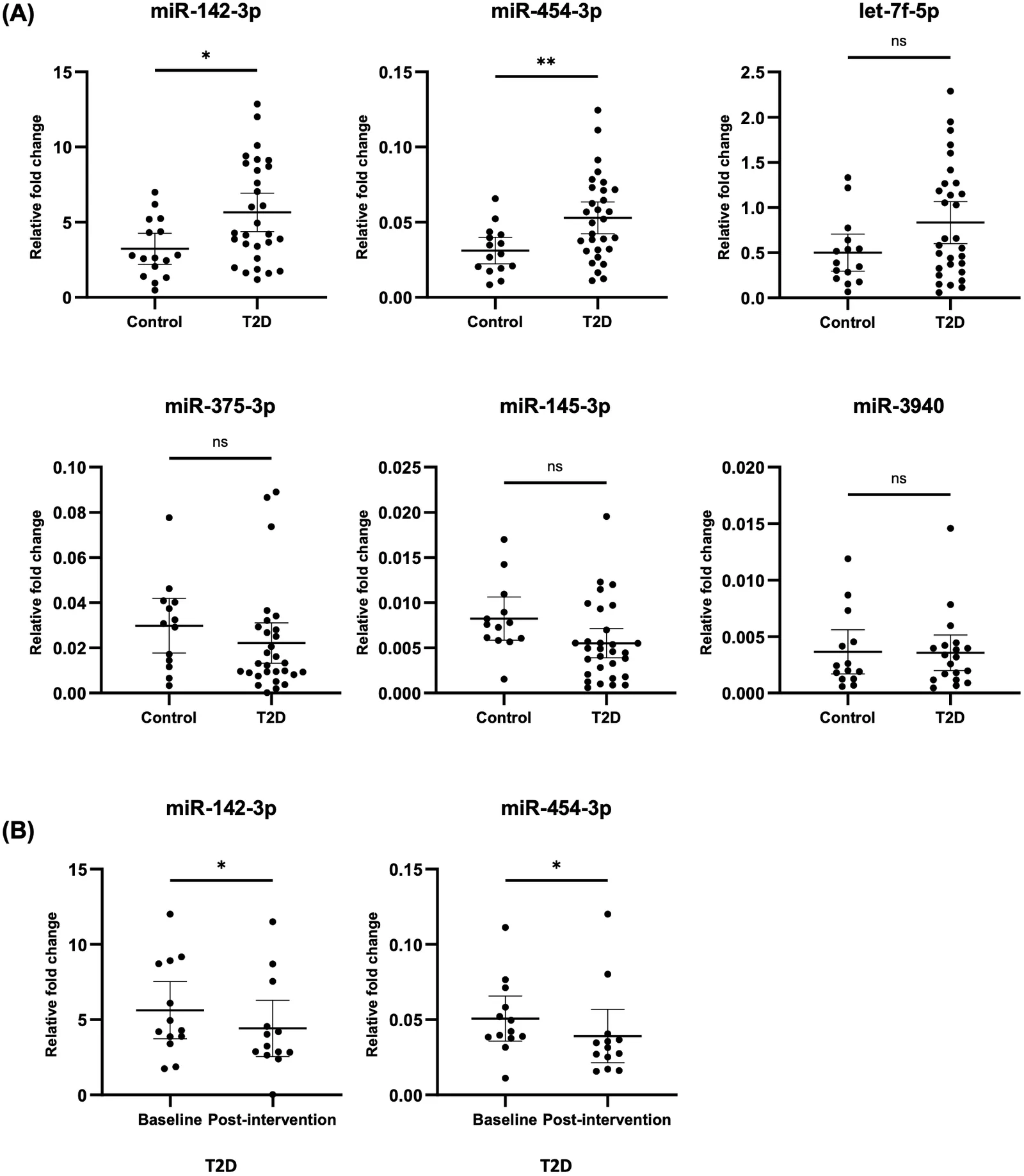
Quantification of sEVs miRNAs expressions by dPCR. **(A)** Based on NGS results, dPCR was performed to determine the expressions of nine miRNAs, miR-142-3p, miR-454-3p, let-7f-5p, miR-375-3p, miR-145-3p, miR-3940, miR-205-3p, miR-205-5p, and miR-1255a. The expressions of miR-205-3p, miR-205-5p, and miR-1255a were below the detection threshold of dPCR, hence, data were not presented. The expressions of miR-142-3p and miR-454-3p were validated for their significant upregulation in plasma sEVs of T2D as compared to control group (*p*<0.05, n=45). **(B)** The upregulated sEVs-miRNAs, miR-142-3p and miR-454-3p were evaluated at post-intervention timepoint to study the effects of glycaemic improvements in differential sEVs-miRNAs expression of T2D subjects after 24 weeks of low-GI red rice consumption. The results showed significant downregulation of both miR-142-3p and miR-454-3p in plasma sEVs of T2D subjects at post-intervention as compared to baseline (*p*<0.05, n=13). All values were normalized to miR-191-5p, a reference gene selected based on stability analysis. Data were shown as mean ± 95% confidence interval. *p<0.05, **p<0.01.

Out of the 12 dysregulated miRNAs identified from NGS, only nine miRNAs were further evaluated using dPCR. Three miRNAs were excluded due to high GC content in their sequences. Among them, miR-142-3p and let-7f-5p were the most abundantly expressed miRNAs in plasma sEVs, followed by miR-454-3p, miR-375-3p, miR-145-3p, and miR-3940-3p. The expressions of miR-205-3p, miR-205-5p and miR-1255a were below the detection threshold of dPCR, hence, were excluded from subsequent analysis. Among the six miRNAs, only miR-142-3p and miR-454-3p were validated as significantly upregulated miRNAs in sEVs of T2D subjects as compared to controls. In particular, miR-142-3p was expressed 1.75-fold higher (*p* = 0.011, 95% CI = 0.57-4.27, n=45) and miR-454-3p was expressed 1.7-fold higher (*p* = 0.009, 95% CI = 0.01-0.4, n=45) in the T2D group (**Figure 3A**).

Despite the sex-stratified analysis showing that both miR-142-3p and miR-454-3p were more associated with females (miR-142-3p, *p* = 0.046; miR-454-3p, *p* = 0.034), results from univariate ANOVA analysis confirmed that the interaction was not significant (miR-142-3p, *p* = 0.991; miR-454-3p, *p* = 0.674). On the other hand, no sex-specific effects were observed in the male cohort. Therefore, it can be concluded that the upregulation of sEVs-miR-142-3p and miR-454-3p was not sex-specific.

### 3.5 sEVs-miR-142-3p and miR-454-3p were upregulated in T2D subjects at baseline and decreased following low-GI red rice intervention

Consumption of low-GI red rice was associated with a significant decrease in HbA1c and FPG levels in the subgroup of T2D subjects, indicating improved glycemic control (**Supplementary Table 2**). In line with this glycemic improvement, a parallel reduction in plasma sEVs-miR-142-3p and miR-454-3p levels was also observed in subjects who demonstrated significant reductions in HbA1c and FPG. Notably, the significant reduction in HbA1c (mean difference= -0.8 ± 0.8, *p<*0.001, n=13) and FPG (mean difference= -1.3 ± 2.0, *p* = 0.013, n=13) coincided with the reduction of miR-142-3p (mean difference = −1.2 ± 0.8, 95% CI: −2.24 to −0.18, *p* = 0.025) and miR-454-3p (mean difference = −0.012 ± 0.020, 95% CI: −0.022 to −0.001, *p* = 0.034) between baseline and post-intervention (**Figure 3B**). These findings further supported the roles of sEVs-miRNAs as reliable biomarkers that could reflect changes in T2D conditions.

### 3.6 sEVs-miR-142-3p and miR-454-3p were positively correlated with HbA1c and triglycerides

The correlations between the two sEVs miRNAs, miR-142-3p and miR-454-3p, and other clinically relevant T2D metabolic parameters such as BMI, FPG, HbA1c, total cholesterol, HDL, LDL, triglyceride, and WC were analyzed using Spearman correlation. The results showed that miR-142-3p had a strong positive correlation with miR-454-3p (r=0.814, *p<*0.001, n=45). Both miRNAs were also positively correlated with HbA1c (miR-142-3p r=0.363, *p<*0.05; miR-454-3p r=0.326, *p<*0.05, n=45) and triglycerides (miR-142-3p r=0.414, *p<*0.01; miR-454-3p r=0.300, *p<*0.05, n=45). On the other hand, no significant correlation was found between both miRNAs and BMI, FPG, total cholesterol, HDL, and LDL (**Supplementary Figure 1**).

### 3.7 The combination of sEVs-miRNA, waist circumference, and BMI demonstrated high diagnostic values for T2D

**Figure 4:**
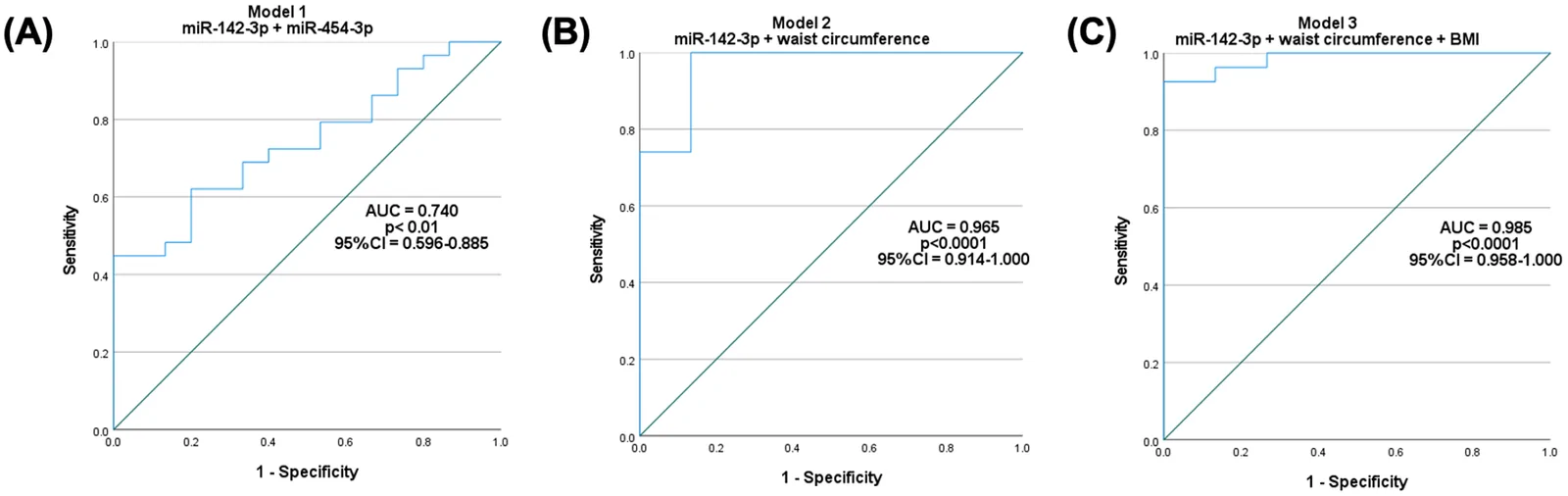
Receiving operating characteristics (ROC) curve for the diagnostic performance of different models to distinguish T2D from control group (n=45). The ROC curves were constructed based on the three models derived from multivariate logistic regressions (model 1 – model 3). **(A)** Model 1: miR-142-3p + miR-454-3p. **(B)** Model 2: miR-142-3p + waist circumference. **(C)** Model 3: miR-142-3p + waist circumference + BMI. Model 3 (miR-142-3p + waist circumference + BMI) was selected as the best diagnostic model to distinguish T2D from control subjects in this study (AUC-ROC=0.985, *p*<0.0001). AUC indicates area under the curve; BMI, body mass index; CI, confidence interval; WC, waist circumference.

To evaluate how well each logistic regression model can classify T2D patients from control subjects, receiver operating characteristic (ROC) curve was generated to evaluate the area under the curve (AUC). Individually, both sEVs miRNAs demonstrated lower discriminative power. The AUC-ROC for miR-142-3p alone was 0.722 (95% CI: 0.570-0.874, *p* = 0.017) and the AUC-ROC for miR-454-3p alone was 0.711 (95% CI: 0.561-0.862, *p* = 0.020). Further evaluation showed that the combination of both miRNAs slightly increased the AUC-ROC to 0.740 (95% CI: 0.596-0.885, *p* = 0.010, **Figure 4A**).

Next, the performance of ROC curve derived from the multivariate logistic regression model was also evaluated. The multivariate logistic regression models were constructed based on the forward likelihood ratio calculations to identify significant variables (**Supplementary Table 5**). Overall, the discriminative power of the ROC curve with multiple parameters had improved as compared to using sEVs-miRNAs alone. In particular, the addition of waist circumference to miR-142-3p increased the AUC-ROC from 0.722 (95% CI: 0.570-0.874, *p* = 0.017) to 0.965 (95% CI: 0.914-1.000, *p<*0.0001; **Figure 4B**). The model’s sensitivity and specificity also improved from 75.9% and 66.7% to 96.3% and 86.7%, respectively. The addition of BMI into the miR-142-3p + waist circumference model further increased its AUC-ROC to 0.985 (95% CI: 0.958-1.000, *p* < 0.001; **Figure 4C**), with the specificity increased to 93.3%. The final model combining miR-142-3p, waist circumference, and BMI achieved an apparent AUC of 0.985. The optimism-corrected AUC was 0.947 (mean optimism = 0.037), indicating minimal overfitting. Therefore, it can be concluded that model 3 (miR-142-3p + WC + BMI) demonstrated the highest diagnostic values to distinguish T2D from the control group, serving as a potential diagnostic model for T2D (**Supplementary Table 6**).

### 3.8 Bioinformatics analysis predicted that the downstream targets of sEVs-miR-142-3p and miR-454-3p were primarily involved in the endocrine system and signal transduction

**Figure 5:**
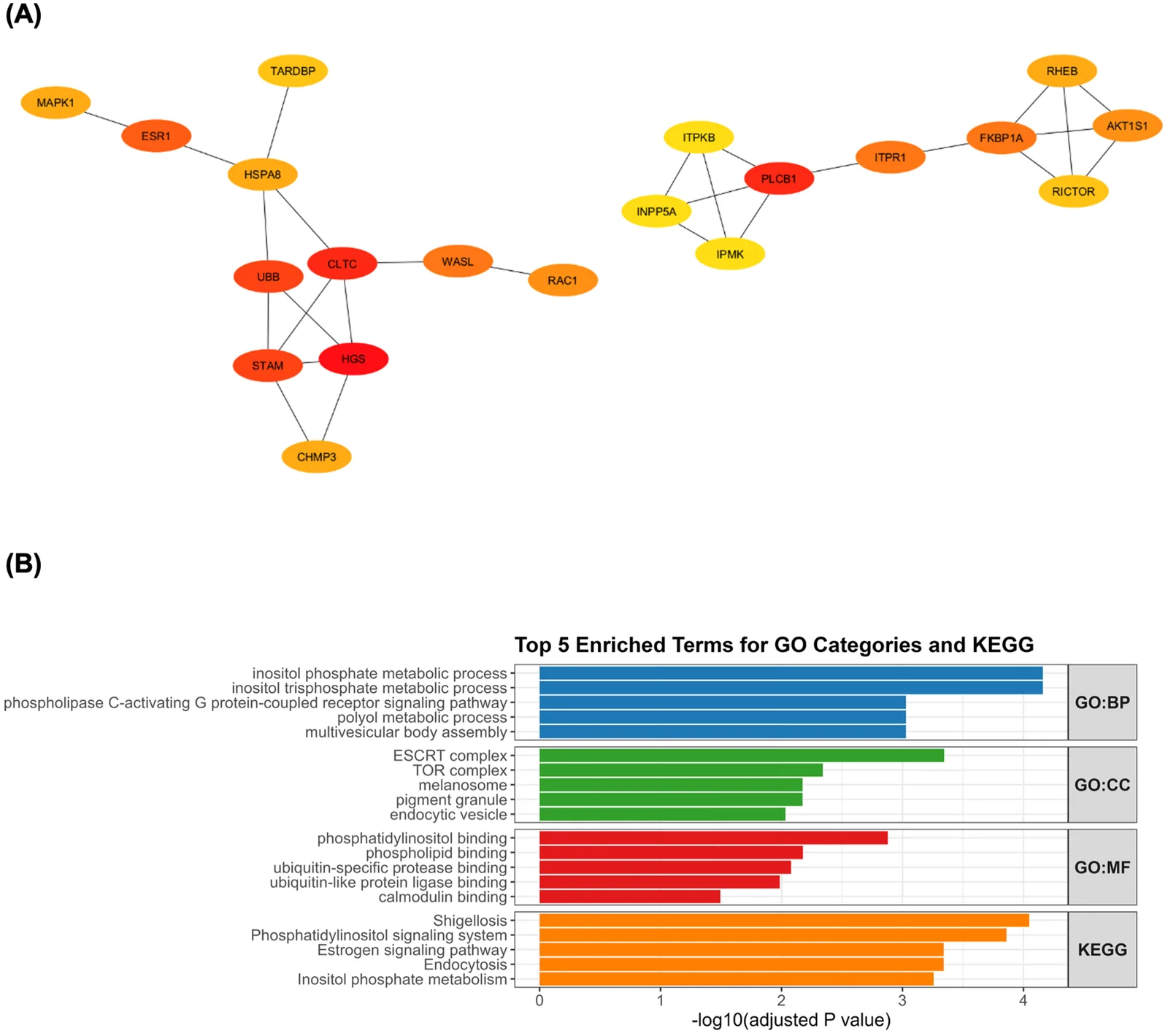
Bioinformatics analyses of upregulated miRNAs (miR-142-3p and miR-454-3p). Protein-protein interactions (PPI) networks analysis identified **(A)** Top 20 hub genes that play central regulatory roles in the PPI network. Topology analysis was performed using the maximum clique centrality algorithm to select the top 20 most connected genes as hub genes from the PPI network. These top 20 hub genes were the most highly connected nodes within the predicted PPI network that were likely to play a pivotal role in T2D development. Further functional analysis conducted using these top 20 hub genes revealed 130, 20, 9, and 74 significantly enriched terms identified in GO-BP, GO-CC, GO-MF, and KEGG, respectively. **(B)** The top 5 enriched terms from GO-BP, GO-CC, GO-MF, and KEGG analyses. These results demonstrated that most of the enriched GO terms and KEGG pathway were closely associated with endocrine system, signal transduction, immune system, and nervous system. BP indicates biological processes; CC, cellular component; MF, molecular function.

The mRNA targets of the two upregulated sEVs-miRNAs, miR-142-3p and miR-454-3p in T2D subjects were predicted using the miRDB, TargetScan, and miRTarbase databases. The results showed that miR-142-3p alone targeted 193 mRNAs, miR-454-3p alone targeted 346 mRNAs, and both the miRNAs had 8 common mRNA targets. These eight common targets were *ZEB2, DYNCLI2, ACBD5, CCDC6, MMGT1, ACSL4, BRWD3, and CFL2* (**Supplementary Figure 2**). To explore the relationship between the mRNA targets from both miRNAs, protein-protein interaction (PPI) network was constructed to identify hub genes that play central roles in regulating the PPI network (**Supplementary Figure 3**). The topology analysis using the maximum clique centrality (MCC) algorithm had selected the top 20 most connected genes as hub genes in this PPI network (**Figure 5A**).

Subsequently, the GO and KEGG pathway analysis was performed on the 20 hub genes to interpret their functions and the relevant biological pathways they were involved in. For GO analysis, target genes were classified according to three commonly reported terms: biological processes (BP), cellular components (CC), and molecular functions (MF). There were 130, 20, and 9 significantly enriched terms identified in BP, CC, and MF categories, respectively. For KEGG analysis, there was a total of 74 significantly enriched pathways identified. Among which, half of the pathways were closely associated with the endocrine system (14/74), signal transduction (9/74), immune system (8/74), and nervous system (6/74) (**Supplementary Tables 7**, **8**). The top 5 GO terms from each category and KEGG enriched terms were presented in **Figure 5B**.

### 3.9 Plasma proteomics profiling revealed linkage between systemically dysregulated pathways and lipid metabolism in T2D

In addition to miRNA analysis, the SomaScan^®^ 11k Assay was performed to identify the key proteins associated with T2D, followed by the gene set enrichment analysis (GSEA) to study the associated hallmarks of functionally related proteins (**Table 4**). In this study, only positively enriched hallmarks were identified with the protein sets. Among which, the “MYC_targets_v1” appeared to be the most significantly enriched hallmark, indicating an association between cell proliferation, cell growth, and T2D (NES = 2.03, FDR<0.0001). Furthermore, the enrichment of several miRNA-predicted pathways such as the “MTORC1 signaling” (NES = 1.77, FDR = 0.002) and “PI3K-AKT-MTOR signaling” (NES = 1.74, FDR = 0.002) hallmarks reinforced the disruption of signal transduction, particularly the EGFR-PI3K-AKT-MTORC1 signaling pathway commonly observed in T2D. Interestingly, the enrichment of hallmarks such as “Reactive oxygen species pathway” (NES = 1.62, FDR = 0.011), “Fatty acid metabolism” (NES = 1.54, FDR = 0.033), “Androgen response” (NES = 1.54, FDR = 0.027), and “Peroxisome” (NES = 1.51, FDR = 0.029) also highlighted the association between oxidative stress, lipid metabolism, and T2D. Lastly, the enrichment of “Complement” (NES = 1.46, FDR = 0.049) suggested the involvement of the innate immune system in T2D.

**Table 4:** Upregulated hallmark gene sets and their leading-edge subset genes identified through the gene set enrichment analysis (GSEA) of proteomic profiles between T2D and control group (n=6).

Hallmarks	Leading-edge genes	Normalized enrichment scores	*p* value	FDR-value
MYC_targets_v1	SMARCC1, CTPS1, CDK2, CCT5, HSPD1, GLO1, EIF3B, CCT2, LDHA, RRM1, EIF2S2, PSMA7, PCBP1, SSBP1, MYC, PRPS2, TCP1, TRA2B, ETF1, EIF4G2, KPNB1, APEX1, HNRNPA2B1, HDAC2, HNRNPD, EIF2S1, RACK1, PGK1, EIF4H, HNRNPA1, HDGF, HSP90AB1, PRDX3, G3BP1, KARS1, NAP1L1, RAD23B, HPRT1, NPM1, UBE2E1, EEF1B2, PSMD8, NME1, RAN, UBA2, SRSF1, COX5A, PCNA, EPRS1, HSPE1, CDC20, SERBP1, PPIA, UBE2L3, ACP1, HNRNPR, EIF3J, SRSF7, PABPC1, CCT7, SRPK1, ERH, SNRPG, PHB2, EIF4E, RANBP1, GOT2, DUT, SNRPB2, and RPS5.	2.03	<0.0001	<0.0001
MTORC1 signaling	GMPS, NFKBIB, PSAT1, CCT6A, SORD, HMGCS1, HMBS, HSPD1, IDH1, SKAP2, GSK3B, LDHA, EIF2S2, IDI1, PPA1, PNP, ETF1, UNG, STIP1, NMT1, PRDX1, GGA2, CACYBP, TPI1, ENO1, PGK1, MTHFD2L, RIT1, TCEA1, PDAP1, HMGCR, NHERF1, MTHFD2, G6PD, HPRT1, GAPDH, DAPP1, STC1, HK2, QDPR, UFM1, GSR, DDIT3, EPRS1, SHMT2, FKBP2, HSPE1, PPIA, CTSC, SSR1, TXNRD1, MAP2K3, BHLHE40, WARS1, HSPA9, ME1, TFRC, CALR, and PLOD2.	1.77	<0.0001	0.002
PI3k/AKT/MTOR signaling	NFKBIB, MAPK9, SMAD2, CDK2, PRKCB, GSK3B, PAK4, RPS6KA3, GRK2, PTPN11, MAPKAP1, AKT1, GRB2, MAPK1, MAPK10, IL2RG, PPP1CA, VAV3, RAC1, RIT1, DAPP1, RAF1, CAB39, PFN1, DDIT3, PLCB1, SFN, TNFRSF1A, YWHAB, IRAK4, EIF4E, PTEN, MKNK1, MYD88, UBE2N, FGF6, RPS6KA1, and MAP2K3	1.74	<0.0001	0.002
Reactive oxygen species pathway	FTL, PFKP, GPX4, FES, OXSR1, PRDX1, GCLM, G6PD, CDKN2D, MBP, PTPA, CAT, PDLIM1, LSP1, GSR, NQO1, TXNRD1, ATOX1, SOD1, GLRX, and MPO	1.62	0.004	0.011
Fatty acid metabolism	ADH1C, SUCLA2, UGDH, MGLL, ACAT2, HMGCS1, HSD17B10, IDH1, ACOX1, METAP1, BLVRA, ACADVL, LDHA, CYP1A1, ACOT8, ERP29, FASN, IDI1, PCBD1, CRYZ, CBR3, HSD17B4, APEX1, HIBCH, HSP90AA1, GPD1, ALDH1A1, ACO2, GLUL, GABARAPL1, MIX23, ACSL4, ALDH3A1, HMGCL, ECHS1, ECI1, RAP1GDS1, MDH2, ECI2, CBR1, HADH, FABP1, DECR1, ACAA1, PSME1, CEL, YWHAH, CD36, MIF, ME1, and PPARA	1.54	0.002	0.033
Androgen response	SORD, VAPA, HMGCS1, RPS6KA3, TPD52, B2M, PDLIM5, IDI1, AKT1, ACTN1, DBI, UBE2I, FKBP5, HMGCR, TMPRSS2, PTK2B, DNAJB9, NGLY1, GSR, HSD17B14, ALDH1A3, PA2G4, PGM3, TNFAIP8, PMEPA1, STK39, MYL12A, and HOMER2	1.54	0.005	0.027
Heme metabolism	FTCD, NEK7, GMPS, ALDH1L1, SDCBP, BPGM, FN3K, HMBS, IGSF3, HEBP1, BLVRA, CA1, ADD2, HAGH, LPIN2, PICALM, EPB41, PSMD9, SYNJ1, SNCA, CLIC2, FOXJ2, ACP5, HDGF, GCLM, TCEA1, PGLS, NFE2L1, CAT, RAD23A, ARHGEF12, and USP15.	1.53	0.001	0.028
Peroxisome	SMARCC1, CTPS1, IDH1, GSTK1, ACOX1, ACOT8, SCP2, IDI1, HSD17B4, PRDX1, ALDH1A1, ACSL4, HMGCL, CAT, SIAH1, ECI2, CTBP1, PABPC1, RXRG, ACAA1, DLG4, CEL, YWHAH, CRABP2, and SOD1	1.51	0.009	0.029
Xenobiotic metabolism	CRP, ADH1C, UGDH, FBP1, ALDH2, ASL, MTHFD1, ACSM1, ARG1, IDH1, PGD, ACOX1, REG1A, LEAP2, DDT, CYP17A1, CYP1A1, PDLIM5, SERPINE1, DCXR, FAH, LPIN2, CYFIP2, ACOX2, NMT1, ACO2, CYB5A, UPP1, GABARAPL1, KARS1, ADH5, HPRT1, ALDH3A1, CAT, F11, PTGR1, HNF4A, GSR, SHMT2, CBR1, ACP1, CCL25, FABP1, TNFRSF1A, CYP2C18, CROT, BLVRB, SPINT2, NQO1, AKR1C2, CD36, TPST1, CASP6, GSTM4, CFB, LONP1, AKR1C3, HMOX1, GCKR, and GSS.	1.48	0.002	0.041
Complement	ACTN2, S100A9, PDGFB, CTSD, C1R, CPM, USP8, SRC, CSRP1, LYN, DGKG, PLAT, VCPIP1, S100A12, KCNIP3, SERPINE1, CASP3, EHD1, GZMK, USP14, FYN, TNFAIP3, GRB2, CTSH, PRCP, JAK2, CXCL1, IRF2, CTSS, MAFF, APOBEC3G, PRDM4, LAP3, PLA2G4A, RAF1, STX4, PFN1, HNF4A, LGALS3, USP15, PLEK, FCN1, PREP, GP9, CTSC, PLAUR, C2, WAS, GCA, HPCAL4, CD40LG, CD36, APOA4, CFB, ME1, ATOX1, CCL5, MSRB1, F2, and PRSS3.	1.46	<0.0001	0.049

These leading-edge genes were the genes that contributed most significantly (FDR<0.05) to the hallmarks that led to the development of T2D.

## 4 Discussion

Due to the growing evidence of how sEVs are involved in intercellular communication between different insulin target sites, our previous study discussed the potential of sEVs, particularly their miRNA cargoes, as biomarkers for T2D diagnosis (39). In this study, the miRNA contents of plasma sEVs isolated from T2D subjects were profiled and compared against non-T2D controls. First, the isolated sEVs were characterized by following the standards of MISEV 2023 (28). In line with previous sEVs studies using diabetes models, our results demonstrated no significant differences in terms of sEV morphology, size distribution, particle concentration, and protein marker expression between T2D and control groups (**Figure 1**) (40, 41). However, it is also noteworthy that a previous study has reported increased circulating sEVs concentrations in T2D subjects (42). These discrepancies may have resulted from differences in study population characteristics or sEV isolation method, which remains poorly standardized across the field.

Indeed, our study identified the upregulation of two miRNAs, miR-142-3p and miR-454-3p, in sEVs of T2D subjects (**Figure 3**). When assessed using the univariate ANOVA analysis, the results showed that there was no significant interaction between sex and sEV-miRNA expressions. Hence, the possible effect of sex on sEV-miRNA expressions were ruled out. Furthermore, this is the first study that demonstrated a concurrent change between sEV-miRNA expressions and improvements in FPG and HbA1c. However, since the causal relationship between these two miRNAs and glycemic measures cannot be independently confirmed, the results should be interpreted as exploratory. Confirmation in a prespecified longitudinal cohort will be needed before this relationship can be established.

To further explore the potential of these sEVs-miRNAs, several diagnostic models were constructed based on the forward likelihood ratio calculations. Among all the models, the combination of sEVs-miR-142-3p, waist circumference, and BMI appeared to be the ideal model to distinguish T2D subjects from controls (**Figure 4**). As it is simple, accessible, and non-invasive to obtain waist circumference and BMI values from patients, these indicators were often used to construct risk prediction models for metabolic diseases. In fact, the combination of waist circumference and BMI was already widely adopted to evaluate obesity-related diseases (43, 44). Yang et al. previously reported that increased waist circumference and BMI were associated with higher risks of developing T2D in obese individuals (45). Furthermore, both parameters were also associated with higher risks of cardiometabolic diseases in T2D patients. Hence, there were certainly added benefits for the combined use of waist circumference and BMI as compared to using only each individual variable. Similarly, the results from this study also demonstrated that the combination of sEVs-miR-142-3p, waist circumference, and BMI achieved the highest sensitivity and specificity values (**Figure 4**). Taken together, these findings suggested the application potential of sEVs-miR-142-3p combined with waist circumference and BMI as biomarkers for T2D diagnosis.

Both miR-142-3p and miR-454-3p are located on the reverse strand of chromosome 17. However, despite being on the same chromosome, they are located far from each other. Therefore, these two miRNAs do not belong to the same cluster (46). In other words, both miRNAs are transcribed under distinct regulatory mechanisms which target different genes and pathways. Therefore, the functional analysis was conducted using the top 20 hub genes identified from the PPI interaction network of miR-142-3p and miR-454-3p, instead of focusing only on the common targets of both miRNAs (**Figure 5A**). Our results from functional analysis identified that the overrepresented pathways were mainly related to the endocrine system and signal transduction (**Supplementary Table 8**). For instance, the proteins related to PI3K/AKT pathways such as the inositol phosphate, inositol triphosphate, and phosphatidylinositol, as well as many lipid-related GO terms such as phospholipids and phospholipase were found to be highly enriched (**Figure 5B**). Within the endocrine system, sEVs-miR-142-3p and miR-454-3p were found to take part in sex hormone regulation, thyroid and parathyroid hormone regulation, as well as blood pressure regulation. These hormones play pivotal roles in the development of T2D as most of them are indirectly involved in causing insulin resistant and maintaining glucose homeostasis. Collectively, these findings from bioinformatics analyses suggested that miR-142-3p and miR-454-3p were intricately involved in the pathways that were responsible for obesity-induced T2D.

Previously, the upregulation of miR-142-3p was found to be associated with increased lipid accumulation within the skeletal muscle cells. Furthermore, findings from lipidomic analysis showed that these lipid droplets were enriched in phosphatidylcholine, the main components in sEV membranes that were involved in EV release and uptake (47, 48). In contrast, overexpression of miR-142-3p in adipocytes was found to suppress adipocytes differentiation and reduce lipid formation in mouse models (49). Such findings suggested that miR-142-3p might have different targets across different cell types, which in turn regulate distinct pathways in lipid homeostasis. On the other hand, the upregulation of miR-454-3p was previously reported to be associated with both T1D and T2D. However, the information on its mRNA targets and associated pathways was very limited (50–53). Thus far, only Liu et al. had identified the *Yy1* gene (encodes for the Yy1 protein) as a target of miR-454-3p in an *in vivo* setting (53). The downregulation of *Yy1* has been linked to mitochondrial dysfunction and lipid accumulation in the liver cells of diabetic mouse model (54, 55).

While previous studies often focused on circulating miRNAs, the unique signatures of miRNAs and their corresponding isoforms in plasma sEVs were often overlooked. In this study, while the majority of miRNAs were identified as the canonical forms of miRNAs, a large repertoire of isomiR variants was also observed (**Table 3**). In particular, the majority of isomiRs identified in T2D patients were 3’ modified (**Figure 2D**). In concordance with this study, Auddino et al. had recently identified that in human β- cells, 3’ end trimming represented the largest isomiR fractions among all isomiRs types, ranging from 55.8% to 71.8% (56). However, another study had otherwise reported that 5’-shifted isomiRs were the key regulators in T2D (57). These distinct findings could be attributed to the use of different cell models and differences in bioinformatics pipelines used for isomiR analysis.

Building upon the findings from miRNA profiling and functional analysis, the proteomics results from this study revealed several dysregulated hallmarks which are commonly involved in the pathogenesis of T2D, including oxidative stress, fatty acid metabolism, and innate immune system activation (**Table 4**). Most of these proteins were involved in the shared pathways regulating T2D and obesity, which further strengthened the linkage between obesity and T2D development. For example, the upregulation of hallmarks such as “MTORC1”, “PI3k/AKT/MTOR signaling”, “Reactive oxygen species pathway”, “Fatty acid metabolism”, and “Peroxisome” was associated with the mechanisms of obesity-induced T2D. Indeed, an excessive amount of fatty acid in the circulation promotes chronic low-grade inflammation, which in turn leads to increased ROS production and peroxisome dysfunction (58, 59). The pro-inflammatory cytokines released by macrophages, such as TNF-α and IL-6, have inhibitory effects on the insulin receptor, IRS1 (60). Consequently, the downstream PI3K-AKT-mTOR signaling pathway is impaired and eventually leads to reduced glucose uptake into body cells (58). Besides that, our results also suggested the involvement of several underappreciated factors such as the roles of the MYC oncogene, steroid hormones, and microbiota in T2D (61–63). Evidently, these systemic dysregulated protein pathways were in concordance with the miRNA target pathways predicted with functional analysis. Together, the integrated results from miRNA prediction analyses and protein profiling provided great insights into the interplay between miRNAs and proteins, suggesting the potential roles of miR-142-3p and miR-454-3p in the development of obesity-induced T2D.

The current study has a few limitations. As compared to the majority of multi-omics clinical studies, this study, which focused on the Malaysian population, has a relatively smaller sample size. The diagnostic performance of the proposed model could be further evaluated in a larger, independent cohort. Furthermore, a large-scale study involving diverse global populations and more omics layers would be useful to gain more insights into the complexity of T2D. Although no study to date has reported significant effects of glucose-lowering and blood-pressure-lowering drugs on sEVs-miR-142-3p and miR-454-3p specifically, potential influence of these medications on sEV-miRNA expressions cannot be completely ruled out. Next, this study focused on circulating sEVs miRNAs and protein expressions. Hence, the tissue-specific sEVs miRNAs and protein expression would not be fully captured. Other technical limitations include the inability to separate AGO-bound miRNAs from free miRNAs within sEVs, as current isolation techniques cannot distinguish between the two populations once they are packaged inside the sEVs. Moreover, plasma lipoproteins such as ApoE and ApoB100 were also not included, hence, the possibility of co-isolated lipoproteins cannot be definitively ruled out. The limitation of isomiR validation from small RNA sequencing, and the limitation of protein profiling to only a customized set of 11k proteins were also acknowledged in this study.

In conclusion, this is the first study that identified a distinct sEV-miRNA signature, particularly the upregulation of miR-142-3p and miR-454-3p in T2D patients within the Malaysian population. Most importantly, improvements in HbA1c and FPG in T2D patients aligned with the downregulation of miR-142-3p and miR-454-3p in their plasma sEVs, indicating that miRNAs could potentially be used as biomarkers for T2D diagnosis. Functional analyses revealed that these miRNAs are primarily involved in pathways associated with insulin resistance and obesity-induced T2D. Furthermore, the combination of sEVs-miR-142-3p, waist circumference, and BMI was demonstrated to have high diagnostic power to distinguish T2D. This study not only profiled sEV-miRNA signatures but also demonstrated the changes in sEVs-miRNAs expression followed by improvements in FPG and HbA1c. Thus, findings from this study will contribute toward the field of translational and precision medicine, providing more insights into the diagnosis, monitoring, and management of T2D.

## Data Availability

The datasets presented in this study can be found in online repositories. The names of the repository/repositories and accession number(s) can be found in the article/**Supplementary Material**.
