# Supplementary material for: Identification of differentially expressed plasma small extracellular vesicles miRNAs as biomarkers for type 2 diabetes
Source: Front Endocrinol (Lausanne). 2026 Sep 9;17:1887230. doi: 10.3389/fendo.2026.1887230 (PMC13597389; doi:10.3389/fendo.2026.1887230)
Supplement: Supplementary file 1 [file SupplementaryFile1.docx]

**Table S1** Summary of candidate reference gene stability values in plasma sEVs samples. The stability values of eight candidate reference genes were first evaluated using four independent algorithms, including GeNorm, NormFinder, BestKeeper, and the comparative Delta Ct method, and their comprehensive stability was then calculated as the geometric mean of the rankings from the four algorithms. A lower geometric mean ranking value indicates greater stability as a reference miRNA. Our results showed that miR-324-3p was ranked as the most stably expressed reference miRNA overall, however, its mean Ct value (31.01) was too high for a reference miRNA. miR-126-3p, on the other hand, was reported to be associated with gestational diabetes and prediabetes. As a result, miR-191-5p was selected as the reference miRNA for normalization in this study.

| **Candidate gene(s)** | **Comprehensive ranking** | **Overall ranking** |
| --- | --- | --- |
| miR-324-5p | 2.45 | 1 |
| miR-191-5p | 2.78 | 2 |
| miR-126-3p | 2.78 | 3 |
| let-7i-5p | 2.83 | 4 |
| cel-miR-39 | 3.35 | 5 |
| miR-16-5p | 4.74 | 6 |
| miR-1-3p | 5.92 | 7 |
| U6 | 8.00 | 8 |

**Table S2** Baseline and post-intervention characteristics of T2D subjects (n=13). T2D subjects were subjected to 24 weeks of low-GI red rice intervention to study the effects of substituting white rice with low-GI red rice on the anthropometry measurements, blood biochemical analysis and small EVs miRNAomes of T2D group. After 24 weeks of low-GI red rice intervention, T2D subjects had significantly lower FPG and HbA1c as compared to baseline.

| **Characteristics** | **T2D (n=13)** | | ***p* value** |
| --- | --- | --- | --- |
|  | **Baseline** | **Post-intervention** |  |
| **Age (year)** | 47.2±8.1 | |  |
| **Sex** |  |  |  |
| Male, n (%) | 6 (46.2) | |  |
| Female, n (%) | 7 (53.8) | |  |
| **Ethnicity** |  |  |  |
| Malay, n (%) | 9 (69.2) | |  |
| Chinese, n (%) | 2 (13.4) | |  |
| Indian, n (%) | 2 (13.4) | |  |
| **BMI (kg/m²)** | 32.0±5.6 | 31.2±6.0 | 0.267 ^a^ |
| **Waist circumference (cm)** | 98.9±13.5 | 96.9±14.8 | 0.111 ^a^ |
| **FPG (mmol/L)** | 8.3±2.3 | 7.0±2.0 | 0.008 ^a**^ |
| **HbA1c (%)** | 8.1±0.9 | 7.3±0.9 | 0.002 ^a**^ |
| **Total cholesterol (mmol/L)** | 5.0±0.9 | 4.9±1.2 | 0.649 ^a^ |
| **HDL-C (mmol/L)** | 1.4±0.5 | 1.4±0.5 | 0.817 ^a^ |
| **LDL-C (mmol/L)** | 2.8±0.7 | 2.8±0.9 | 0.809 ^a^ |
| **Triglycerides (mmol/L)** | 1.7±0.5 | 1.6±0.5 | 0.408 ^a^ |

^a^ Paired sample t-test

Values were presented as mean±SD. BMI indicates body mass index; FPG, fasting plasma glucose; HDL-C, high density lipoprotein; LDL-C, low density lipoprotein; T2D, Type 2 Diabetes. ** p<0.01.

**Table S3** Summary of total RNA concentration and purity for all samples (n = 63). The purity of total RNA was assessed using A260/280 and A260/230 absorbance ratios, which indicate protein and salt/organic solvent contamination, respectively.

| **A260/A280** | **A260/230** | **Concentration (ng/uL)** |
| --- | --- | --- |
| 1.47 ± 0.16 | 0.55 ± 0.49 | 18.5 ± 9.3 |

**Table S4** Differentially expressed sEVs-isomiRs between T2D and control group (n=36). A total of 94 isomiRs were dysregulated (43 upregulated and 51 downregulated). IsomiRs were considered as differentially expressed when |log2fold change|>1 and FDR<0.05.

| **Name** | **Log₂ fold change** | ***p* value** | **FDR value** |
| --- | --- | --- | --- |
| **Upregulated** | | | |
| hsa-miR-142-3p | 1.244 | <0.0001 | 0.004 |
| hsa-miR-15a-5p.G | 2.026 | <0.0001 | 0.009 |
| hsa-let-7a-5p.G5T | 2.135 | <0.0001 | 0.048 |
| hsa-miR-144-3p.ts.ct | 2.208 | <0.0001 | 0.023 |
| hsa-miR-1-3p.T20G.T22G | 2.303 | <0.0001 | 0.048 |
| hsa-let-7f-5p.As.t | 2.925 | <0.0001 | 0.029 |
| hsa-miR-320a-3p.T12G | 2.955 | <0.0001 | 0.031 |
| hsa-miR-100-5p.T | 3.320 | <0.0001 | 0.034 |
| hsa-miR-320a-3p.T12G.a | 3.455 | <0.0001 | 0.013 |
| hsa-miR-320a-3p.T12G.A.T | 3.803 | <0.0001 | 0.014 |
| hsa-miR-744-5p.A.G | 3.813 | <0.0001 | 0.034 |
| hsa-miR-1-3p.t | 3.851 | <0.0001 | 0.006 |
| hsa-miR-4508.gs.g | 3.915 | <0.0001 | 0.014 |
| hsa-miR-1-3p.T20G.t | 3.932 | <0.0001 | 0.031 |
| hsa-miR-320b.A22T.TA | 4.031 | <0.0001 | 0.023 |
| hsa-miR-93-3p.T6G.T | 4.220 | <0.0001 | 0.038 |
| hsa-miR-652-5p.As.C24T.A25T | 4.657 | <0.0001 | 0.031 |
| hsa-miR-1276.C | 4.681 | <0.0001 | 0.038 |
| hsa-miR-28-3p.Cs | 4.708 | <0.0001 | 0.032 |
| hsa-miR-4492.gs.G | 4.828 | <0.0001 | 0.048 |
| hsa-miR-184.T22A | 5.160 | <0.0001 | 0.034 |
| hsa-miR-544b.aa | 5.254 | <0.0001 | 0.004 |
| hsa-miR-4433b-3p.As.G8C.AA | 5.487 | <0.0001 | 0.024 |
| hsa-miR-4433b-3p.G8C.A | 5.590 | <0.0001 | 0.015 |
| hsa-miR-137-3p.A10G.G18A.ag | 5.663 | <0.0001 | 0.024 |
| hsa-miR-4433b-3p.G8C.t | 5.774 | <0.0001 | 0.014 |
| hsa-miR-184.C12T.G21A.t | 6.025 | <0.0001 | 0.014 |
| hsa-miR-4433b-3p.G8C.AC | 6.054 | <0.0001 | 0.006 |
| hsa-miR-4433b-3p.G8T | 6.221 | <0.0001 | 0.013 |
| hsa-miR-1-3p.A14G.T20C.t | 6.357 | <0.0001 | 0.001 |
| hsa-miR-184.t | 6.516 | <0.0001 | <.0001 |
| hsa-miR-1-3p.A17T.A21G.t, hsa-miR-206.G11A.G17T.g | 6.520 | <0.0001 | 0.004 |
| hsa-miR-4433b-3p.As.G8C.AT | 6.707 | <0.0001 | 0.001 |
| hsa-miR-4433b-3p.As.G8C.A | 6.812 | <0.0001 | 0.001 |
| hsa-miR-4433b-3p.As.G8T | 7.198 | <0.0001 | 0.001 |
| hsa-miR-4433b-3p.As.G8C.t | 7.331 | <0.0001 | <0.0001 |
| hsa-miR-4433b-3p.G8C.T | 7.535 | <0.0001 | <0.0001 |
| hsa-miR-100-5p.TT | 7.613 | <0.0001 | <0.0001 |
| hsa-miR-4433b-3p.As.G8C.T | 7.779 | <0.0001 | <0.0001 |
| hsa-miR-4433b-3p.G8C.AT | 7.825 | <0.0001 | <0.0001 |
| hsa-miR-1-3p.T22G | 8.731 | <0.0001 | <0.0001 |
| hsa-miR-4433b-3p.G8C | 9.677 | <0.0001 | <0.0001 |
| hsa-miR-4433b-3p.As.G8C | 10.956 | <0.0001 | <0.0001 |
| **Downregulated** | | | |
| hsa-miR-486-5p.T9G.G22A.G | -5.789 | <0.0001 | 0.013 |
| hsa-miR-320a-3p.Cs.A22C | -5.745 | <0.0001 | 0.013 |
| hsa-miR-483-5p.G22A.TT | -5.541 | <0.0001 | 0.014 |
| hsa-miR-92b-5p.G22A | -5.350 | <0.0001 | 0.024 |
| hsa-miR-151a-3p.G20A.g | -5.339 | <0.0001 | 0.014 |
| hsa-miR-486-5p.T9G.C19T | -5.098 | <0.0001 | 0.037 |
| hsa-miR-192-5p.AT | -5.030 | <0.0001 | 0.004 |
| hsa-miR-6823-5p.TT | -4.954 | <0.0001 | 0.036 |
| hsa-miR-6833-5p.gs.a | -4.907 | <0.0001 | 0.045 |
| hsa-miR-3129-5p.t | -4.815 | <0.0001 | 0.043 |
| hsa-miR-642a-3p.ags.T | -4.792 | <0.0001 | 0.045 |
| hsa-miR-4669.ts.g | -4.720 | <0.0001 | 0.034 |
| hsa-miR-122-5p.G5A.G22T | -4.358 | <0.0001 | 0.013 |
| hsa-miR-432-5p.G11A.gg | -4.353 | <0.0001 | 0.007 |
| hsa-miR-24-3p.C20A.G22A.G | -4.311 | <0.0001 | 0.048 |
| hsa-miR-320d.Gs.A.T | -4.110 | <0.0001 | 0.005 |
| hsa-miR-770-5p.C | -3.751 | <0.0001 | 0.039 |
| hsa-miR-3064-5p.tcs | -3.670 | <0.0001 | 0.014 |
| hsa-miR-25-3p.AA | -3.501 | <0.0001 | 0.036 |
| hsa-miR-187-5p.gs.G | -3.454 | <0.0001 | 0.006 |
| hsa-miR-143-3p.ts.A8G | -3.060 | <0.0001 | 0.048 |
| hsa-miR-143-3p.ts.A11G | -3.054 | <0.0001 | 0.014 |
| hsa-miR-143-3p.G18A.T | -3.018 | <0.0001 | 0.034 |
| hsa-miR-197-3p.C15T | -3.018 | <0.0001 | 0.023 |
| hsa-miR-143-3p.C19T.T | -2.762 | <0.0001 | 0.034 |
| hsa-miR-143-3p.A5G.A | -2.572 | <0.0001 | 0.034 |
| hsa-miR-145-3p.ggs.T | -2.317 | <0.0001 | 0.037 |
| hsa-miR-423-5p.G14C.tt | -2.286 | <0.0001 | 0.028 |
| hsa-miR-122-5p.ts.AA | -2.275 | <0.0001 | 0.048 |
| hsa-miR-143-3p.C13T | -2.261 | <0.0001 | 0.048 |
| hsa-miR-205-5p.g | -2.219 | <0.0001 | 0.010 |
| hsa-miR-143-3p.ts | -2.185 | <0.0001 | 0.034 |
| hsa-miR-375-3p.ts.a | -2.130 | <0.0001 | 0.002 |
| hsa-miR-375-3p.ga | -1.914 | <0.0001 | 0.006 |
| hsa-miR-197-3p.A7G | -1.763 | <0.0001 | 0.048 |
| hsa-miR-486-5p.C19A.T | -1.763 | <0.0001 | 0.048 |
| hsa-miR-143-3p.A5G | -1.726 | <0.0001 | 0.014 |
| hsa-miR-125b-5p.ga | -1.707 | <0.0001 | 0.004 |
| hsa-miR-375-3p.a | -1.703 | <0.0001 | 0.007 |
| hsa-miR-143-3p.tgs | -1.694 | <0.0001 | 0.048 |
| hsa-miR-143-3p.A12G | -1.688 | 0.0001 | 0.034 |
| hsa-miR-375-3p | -1.602 | 0.0002 | 0.048 |
| hsa-miR-143-3p.T | -1.597 | <0.0001 | 0.013 |
| hsa-miR-3940-3p.t | -1.593 | <0.0001 | 0.048 |
| hsa-miR-143-3p | -1.501 | <0.0001 | 0.034 |
| hsa-miR-143-3p.C21T | -1.394 | <0.0001 | 0.034 |
| hsa-miR-627-5p.gs.GT | -1.375 | 0.0001 | 0.034 |
| hsa-miR-486-5p.G22A.G.A | -1.156 | 0.0002 | 0.048 |
| hsa-miR-30e-3p.C22A | -1.095 | 0.0001 | 0.034 |
| hsa-miR-29c-3p.ta | -1.059 | 0.0001 | 0.048 |
| hsa-miR-27a-3p.c | -1.032 | 0.0001 | 0.038 |

**Figure S1** Correlation between sEVs miRNAs, BMI, FPG, HbA1c, TC, HDL-C, LDL-C, and TG in T2D subjects (n=45). Interestingly, significant positive correlations were observed between miR-142-3p and miR-454-3p, as well as between both miRNAs with TG and HbA1c, the clinical gold standard for long term glycaemic control. The red lines represent the linear regression trendlines and grey areas represent 95% confidence interval. BMI indicates body mass index; FBG, fasting blood glucose; HbA1c, glycated haemoglobin; TC, total cholesterol; HDL, high density lipoprotein; LDL, low density lipoprotein; TG, triglyceride; WC, waist circumference. *p<0.05, ***p<0.001.

**Table S5** Multivariate logistic regression analysis assessing different models consisted of sEVs miRNAs and T2D risk factors. Model 1 which consisted of two sEVs miRNAs showed extremely high odds ratio value and wide range of 95% CI, suggesting model instability. To derive more robust models for clinical interpretation, the forward likelihood ratio (LR) logistic regression was performed. The final calculations showed that only miR-142-3p, waist circumference, and BMI were independently and significantly associated with T2D (model 2 & 3).

| **Parameter** | **Omnibus test** | **Hosmer-Lemeshow** | **Exp(B) (Odds ratio)** | **95% CI** | | ***p* value** | **Nagelkerke R²** |
| --- | --- | --- | --- | --- | --- | --- | --- |
|  |  |  |  | **lower** | **upper** |  |  |
| **Model 1: miR-142-3p + miR-454-3p** | | | | | | | |
| miR-142-3p | 0.011 | 0.698 | 1.337 | 0.825 | 2.165 | 0.238 | 0.259 |
| miR-454-3p |  |  | 4685280 | 0 | 2.39E+30 | 0.581 |  |
| **Model 2: miR-142-3p + WC** | | | | | | | |
| miR-142-3p | <0.001 | 0.186 | 2.675 | 1.069 | 1.698 | 0.036 | 0.805 |
| WC |  |  | 1.277 | 1.07 | 1.522 | 0.007 |  |
| **Model 3: miR-142-3p + WC + BMI** | | | | | | | |
| miR-142-3p | <0.001 | 0.986 | 5.028 | 1.123 | 22.517 | 0.035 | 0.868 |
| WC |  |  | 1.271 | 1.014 | 1.592 | 0.038 |  |
| BMI |  |  | 1.971 | 0.932 | 4.169 | 0.076 |  |

**Table S6** Summary of receiving operating characteristics (ROC) analysis of multivariate logistic regression models. The ROC curves were constructed based on the three models derived from the forward LR logistic regressions calculation (model 1–model 3). Model 3 (miR-142-3p + waist circumference + BMI) was selected as the best diagnostic model to distinguish T2D from control subjects in this study as it demonstrated both high sensitivity and specificity (AUC= .985, p<0.0001). The cut-off values were determined based on highest Youden’s index (sensitivity+specificity-1). AUC indicates area under curve; BMI, body mass index; CI, confidence interval; FPG, fasting plasma glucose; WC, waist circumference.

| **AUC** | ***P* value** | **95% CI** | **Sensitivity** | **Specificity** | **Cut-off** | **Youden's index** |
| --- | --- | --- | --- | --- | --- | --- |
| ***Model 1: miR-142-3p + miR-454-3p*** | | | | | | |
| 0.740 | 0.01 | 0.596-0.885 | 0.621 | 0.800 | 0.661 | 0.421 |
| ***Model 2: miR-142-3p + WC*** | | | | | | |
| 0.965 | <0.0001 | 0.914-1.000 | 0.963 | 0.867 | 0.438 | 0.830 |
| ***Model 3: miR-142-3p + WC + BMI*** | | | | | | |
| 0.985 | <0.0001 | 0.958-1.000 | 0.926 | 0.933 | 0.664 | 0.859 |

**Figure S2** Overview of miRNA-mRNA gene interaction network of the upregulated miRNAs (miR-142-3p and miR-454-3p). The interaction network was constructed using common mRNAs targets predicted by TargetScan, miRTarbase, and miRDB databases. 193 mRNAs were the target for miR-142-3p, 346 mRNAs were the target for miR-454-3p. There were 8 common targets of both miRNAs, including ZEB2, DYNCLI2, ACBD5, CCDC6, MMGT1, ACSL4, BRWD3, and CFL2.

**Figure S3** Protein-protein interaction (PPI) network of mRNA targets. To explore the relationship between the mRNA targets from both miRNAs, protein-protein interaction (PPI) network was constructed to identify hub genes that played important roles in the regulatory network. The initial PPI network obtained from STRING showed a total of 346 nodes and 109 edges, however, 206 nodes were discarded as they were not connected to the main PPI network. As a result, the final PPI network was consisted of 140 nodes and 200 edges, with the average node degree of 2.86. From this PPI network, the top 20 hub genes were extracted to perform functional enrichment analysis.

**Table S7** Significantly enriched Gene Ontology (GO) terms of top 20 hub genes identified from PPI network. The results from functional enrichment analysis were categorized into Biological Processes (BP), Cellular Components (CC), and Molecular Function (MF). The total significant enriched GO terms for BP, CC, and MF were 130, 20, and 9, respectively.

|  | **Description** | **Gene Ratio** | **Bg Ratio** | **Rich Factor** | **Fold Enrichment** | **zScore** | ***p* value** | ***p* adjust** | **GeneID** |
| --- | --- | --- | --- | --- | --- | --- | --- | --- | --- |
| **Biological processes (BP)** | | | | | | | | | |
| GO:0043647 | inositol phosphate metabolic process | 4/20 | 42/18888 | 0.095 | 89.94 | 18.7871 | <0.001 | <0.001 | INPP5A/PLCB1/IPMK/ITPKB |
| GO:0032957 | inositol trisphosphate metabolic process | 3/20 | 10/18888 | 0.3 | 283.32 | 29.0735 | <0.001 | <0.001 | PLCB1/IPMK/ITPKB |
| GO:0007200 | phospholipase C-activating G protein-coupled receptor signaling pathway | 4/20 | 102/18888 | 0.039 | 37.04 | 11.8808 | <0.001 | 0.001 | ITPR1/INPP5A/PLCB1/ESR1 |
| GO:0019751 | polyol metabolic process | 4/20 | 107/18888 | 0.037 | 35.30 | 11.5856 | <0.001 | 0.001 | INPP5A/PLCB1/IPMK/ITPKB |
| GO:0036258 | multivesicular body assembly | 3/20 | 31/18888 | 0.097 | 91.39 | 16.3989 | <0.001 | 0.001 | CHMP3/HGS/STAM |
| GO:0036257 | multivesicular body organization | 3/20 | 32/18888 | 0.094 | 88.54 | 16.1353 | <0.001 | 0.001 | CHMP3/HGS/STAM |
| GO:0043162 | ubiquitin-dependent protein catabolic process via the multivesicular body sorting pathway | 3/20 | 37/18888 | 0.081 | 76.57 | 14.9808 | <0.001 | 0.001 | CHMP3/HGS/STAM |
| GO:0045324 | late endosome to vacuole transport | 3/20 | 37/18888 | 0.081 | 76.57 | 14.9808 | <0.001 | 0.001 | CHMP3/HGS/STAM |
| GO:1903532 | positive regulation of secretion by cell | 5/20 | 288/18888 | 0.017 | 16.40 | 8.5719 | <0.001 | 0.001 | TARDBP/ITPR1/PLCB1/HGS/STAM |
| GO:0016197 | endosomal transport | 5/20 | 289/18888 | 0.017 | 16.34 | 8.5553 | <0.001 | 0.001 | CHMP3/CLTC/HGS/STAM/MAPK1 |
| GO:0032509 | endosome transport via multivesicular body sorting pathway | 3/20 | 41/18888 | 0.073 | 69.10 | 14.2124 | <0.001 | 0.001 | CHMP3/HGS/STAM |
| GO:0090148 | membrane fission | 3/20 | 44/18888 | 0.068 | 64.39 | 13.7057 | <0.001 | 0.001 | CHMP3/HGS/STAM |
| GO:0051047 | positive regulation of secretion | 5/20 | 312/18888 | 0.016 | 15.13 | 8.1963 | <0.001 | 0.001 | TARDBP/ITPR1/PLCB1/HGS/STAM |
| GO:0071985 | multivesicular body sorting pathway | 3/20 | 48/18888 | 0.063 | 59.03 | 13.1048 | <0.001 | 0.001 | CHMP3/HGS/STAM |
| GO:0046488 | phosphatidylinositol metabolic process | 4/20 | 164/18888 | 0.024 | 23.03 | 9.2268 | <0.001 | 0.002 | INPP5A/PLCB1/IPMK/ITPKB |
| GO:0007034 | vacuolar transport | 4/20 | 171/18888 | 0.023 | 22.09 | 9.0202 | <0.001 | 0.002 | CHMP3/HGS/STAM/HSPA8 |
| GO:0072666 | establishment of protein localization to vacuole | 3/20 | 65/18888 | 0.046 | 43.59 | 11.1977 | <0.001 | 0.003 | HGS/STAM/HSPA8 |
| GO:0002685 | regulation of leukocyte migration | 4/20 | 230/18888 | 0.017 | 16.42 | 7.6625 | <0.001 | 0.005 | WASL/PLCB1/RAC1/MAPK1 |
| GO:0032024 | positive regulation of insulin secretion | 3/20 | 86/18888 | 0.035 | 32.94 | 9.6666 | <0.001 | 0.005 | TARDBP/ITPR1/PLCB1 |
| GO:0031669 | cellular response to nutrient levels | 4/20 | 239/18888 | 0.017 | 15.81 | 7.4996 | <0.001 | 0.005 | RHEB/MAPK1/HSPA8/RICTOR |
| GO:0072665 | protein localization to vacuole | 3/20 | 88/18888 | 0.034 | 32.20 | 9.5496 | <0.001 | 0.005 | HGS/STAM/HSPA8 |
| GO:0051656 | establishment of organelle localization | 5/20 | 472/18888 | 0.011 | 10.00 | 6.4499 | <0.001 | 0.005 | CHMP3/WASL/HGS/UBB/STAM |
| GO:0043328 | protein transport to vacuole involved in ubiquitin-dependent protein catabolic process via the multivesicular body sorting pathway | 2/20 | 15/18888 | 0.133 | 125.92 | 15.7576 | <0.001 | 0.005 | HGS/STAM |
| GO:1903543 | positive regulation of exosomal secretion | 2/20 | 15/18888 | 0.133 | 125.92 | 15.7576 | <0.001 | 0.005 | HGS/STAM |
| GO:0007032 | endosome organization | 3/20 | 97/18888 | 0.031 | 29.21 | 9.0682 | <0.001 | 0.006 | CHMP3/HGS/STAM |
| GO:0006898 | receptor-mediated endocytosis | 4/20 | 259/18888 | 0.015 | 14.59 | 7.1674 | <0.001 | 0.006 | WASL/CLTC/HGS/RAC1 |
| GO:1903541 | regulation of exosomal secretion | 2/20 | 17/18888 | 0.118 | 111.11 | 14.7867 | <0.001 | 0.006 | HGS/STAM |
| GO:0031668 | cellular response to extracellular stimulus | 4/20 | 269/18888 | 0.015 | 14.04 | 7.0148 | <0.001 | 0.007 | RHEB/MAPK1/HSPA8/RICTOR |
| GO:2000641 | regulation of early endosome to late endosome transport | 2/20 | 19/18888 | 0.105 | 99.41 | 13.9726 | <0.001 | 0.007 | CHMP3/MAPK1 |
| GO:1990182 | exosomal secretion | 2/20 | 20/18888 | 0.1 | 94.44 | 13.6119 | <0.001 | 0.007 | HGS/STAM |
| GO:0051258 | protein polymerization | 4/20 | 287/18888 | 0.014 | 13.16 | 6.7597 | <0.001 | 0.007 | CHMP3/WASL/RAC1/RICTOR |
| GO:0090277 | positive regulation of peptide hormone secretion | 3/20 | 111/18888 | 0.027 | 25.52 | 8.4368 | <0.001 | 0.007 | TARDBP/ITPR1/PLCB1 |
| GO:0002793 | positive regulation of peptide secretion | 3/20 | 113/18888 | 0.027 | 25.07 | 8.3561 | <0.001 | 0.007 | TARDBP/ITPR1/PLCB1 |
| GO:0007213 | G protein-coupled acetylcholine receptor signaling pathway | 2/20 | 21/18888 | 0.095 | 89.94 | 13.2771 | <0.001 | 0.007 | ITPR1/PLCB1 |
| GO:0097734 | extracellular exosome biogenesis | 2/20 | 21/18888 | 0.095 | 89.94 | 13.2771 | <0.001 | 0.007 | HGS/STAM |
| GO:0140112 | extracellular vesicle biogenesis | 2/20 | 23/18888 | 0.087 | 82.12 | 12.6738 | <0.001 | 0.008 | HGS/STAM |
| GO:0006650 | glycerophospholipid metabolic process | 4/20 | 307/18888 | 0.013 | 12.30 | 6.5018 | <0.001 | 0.008 | INPP5A/PLCB1/IPMK/ITPKB |
| GO:0071675 | regulation of mononuclear cell migration | 3/20 | 123/18888 | 0.024 | 23.03 | 7.9819 | <0.001 | 0.009 | WASL/PLCB1/MAPK1 |
| GO:0071676 | negative regulation of mononuclear cell migration | 2/20 | 25/18888 | 0.08 | 75.55 | 12.1439 | <0.001 | 0.009 | WASL/PLCB1 |
| GO:0051209 | release of sequestered calcium ion into cytosol | 3/20 | 129/18888 | 0.023 | 21.96 | 7.7781 | <0.001 | 0.009 | FKBP1A/ITPR1/PLCB1 |
| GO:0051283 | negative regulation of sequestering of calcium ion | 3/20 | 130/18888 | 0.023 | 21.79 | 7.7455 | <0.001 | 0.009 | FKBP1A/ITPR1/PLCB1 |
| GO:0032958 | inositol phosphate biosynthetic process | 2/20 | 26/18888 | 0.077 | 72.65 | 11.9020 | <0.001 | 0.009 | IPMK/ITPKB |
| GO:0033598 | mammary gland epithelial cell proliferation | 2/20 | 26/18888 | 0.077 | 72.65 | 11.9020 | <0.001 | 0.009 | ESR1/MAPK1 |
| GO:0051282 | regulation of sequestering of calcium ion | 3/20 | 132/18888 | 0.023 | 21.46 | 7.6813 | <0.001 | 0.009 | FKBP1A/ITPR1/PLCB1 |
| GO:0032511 | late endosome to vacuole transport via multivesicular body sorting pathway | 2/20 | 27/18888 | 0.074 | 69.96 | 11.6735 | <0.001 | 0.009 | HGS/STAM |
| GO:0042026 | protein refolding | 2/20 | 27/18888 | 0.074 | 69.96 | 11.6735 | <0.001 | 0.009 | FKBP1A/HSPA8 |
| GO:0071496 | cellular response to external stimulus | 4/20 | 335/18888 | 0.012 | 11.28 | 6.1786 | <0.001 | 0.009 | RHEB/MAPK1/HSPA8/RICTOR |
| GO:0007041 | lysosomal transport | 3/20 | 136/18888 | 0.022 | 20.83 | 7.5571 | <0.001 | 0.009 | CHMP3/HGS/HSPA8 |
| GO:0051208 | sequestering of calcium ion | 3/20 | 136/18888 | 0.022 | 20.83 | 7.5571 | <0.001 | 0.009 | FKBP1A/ITPR1/PLCB1 |
| GO:0016236 | macroautophagy | 4/20 | 343/18888 | 0.012 | 11.01 | 6.0933 | <0.001 | 0.009 | CHMP3/RHEB/HGS/STAM |
| GO:0046887 | positive regulation of hormone secretion | 3/20 | 144/18888 | 0.021 | 19.68 | 7.3239 | <0.001 | 0.01 | TARDBP/ITPR1/PLCB1 |
| GO:0032006 | regulation of TOR signaling | 3/20 | 145/18888 | 0.021 | 19.54 | 7.2961 | 0.001 | 0.01 | RHEB/AKT1S1/RICTOR |
| GO:0050714 | positive regulation of protein secretion | 3/20 | 147/18888 | 0.02 | 19.27 | 7.2413 | 0.001 | 0.01 | TARDBP/ITPR1/PLCB1 |
| GO:1990806 | ligand-gated ion channel signaling pathway | 2/20 | 31/18888 | 0.065 | 60.93 | 10.8721 | 0.001 | 0.01 | ITPR1/PLCB1 |
| GO:0032535 | regulation of cellular component size | 4/20 | 370/18888 | 0.011 | 10.21 | 5.8248 | 0.001 | 0.011 | CHMP3/AKT1S1/RAC1/RICTOR |
| GO:0006622 | protein targeting to lysosome | 2/20 | 33/18888 | 0.061 | 57.24 | 10.5268 | 0.001 | 0.011 | HGS/HSPA8 |
| GO:0016050 | vesicle organization | 4/20 | 371/18888 | 0.011 | 10.18 | 5.8154 | 0.001 | 0.011 | CHMP3/WASL/HGS/STAM |
| GO:0006066 | alcohol metabolic process | 4/20 | 373/18888 | 0.011 | 10.13 | 5.7967 | 0.001 | 0.011 | INPP5A/PLCB1/IPMK/ITPKB |
| GO:0070507 | regulation of microtubule cytoskeleton organization | 3/20 | 160/18888 | 0.019 | 17.71 | 6.9097 | 0.001 | 0.012 | CHMP3/CLTC/RAC1 |
| GO:0006644 | phospholipid metabolic process | 4/20 | 383/18888 | 0.01 | 9.86 | 5.7053 | 0.001 | 0.012 | INPP5A/PLCB1/IPMK/ITPKB |
| GO:0031929 | TOR signaling | 3/20 | 165/18888 | 0.018 | 17.17 | 6.7924 | 0.001 | 0.013 | RHEB/AKT1S1/RICTOR |
| GO:0030041 | actin filament polymerization | 3/20 | 167/18888 | 0.018 | 16.97 | 6.7469 | 0.001 | 0.013 | WASL/RAC1/RICTOR |
| GO:0050796 | egulation of insulin secretion | 3/20 | 167/18888 | 0.018 | 16.97 | 6.7469 | 0.001 | 0.013 | TARDBP/ITPR1/PLCB1 |
| GO:0050900 | leukocyte migration | 4/20 | 396/18888 | 0.01 | 9.54 | 5.5913 | 0.001 | 0.013 | WASL/PLCB1/RAC1/MAPK1 |
| GO:0046486 | glycerolipid metabolic process | 4/20 | 400/18888 | 0.01 | 9.44 | 5.5573 | 0.001 | 0.013 | INPP5A/PLCB1/IPMK/ITPKB |
| GO:0006458 | 'de novo' protein folding | 2/20 | 41/18888 | 0.049 | 46.07 | 9.4054 | 0.001 | 0.015 | FKBP1A/HSPA8 |
| GO:0060236 | regulation of mitotic spindle organization | 2/20 | 42/18888 | 0.048 | 44.97 | 9.2879 | 0.001 | 0.015 | CHMP3/CLTC |
| GO:0095500 | acetylcholine receptor signaling pathway | 2/20 | 42/18888 | 0.048 | 44.97 | 9.2879 | 0.001 | 0.015 | ITPR1/PLCB1 |
| GO:1990000 | amyloid fibril formation | 2/20 | 42/18888 | 0.048 | 44.97 | 9.2879 | 0.001 | 0.015 | TARDBP/FKBP1A |
| GO:0097553 | calcium ion transmembrane import into cytosol | 3/20 | 187/18888 | 0.016 | 15.15 | 6.3314 | 0.001 | 0.016 | FKBP1A/ITPR1/PLCB1 |
| GO:0045022 | early endosome to late endosome transport | 2/20 | 44/18888 | 0.046 | 42.93 | 9.0650 | 0.001 | 0.016 | CHMP3/MAPK1 |
| GO:1905145 | cellular response to acetylcholine | 2/20 | 44/18888 | 0.046 | 42.93 | 9.0650 | 0.001 | 0.016 | ITPR1/PLCB1 |
| GO:0051223 | regulation of protein transport | 4/20 | 440/18888 | 0.009 | 8.59 | 5.2416 | 0.001 | 0.016 | TARDBP/ITPR1/PLCB1/HSPA8 |
| GO:0071763 | nuclear membrane organization | 2/20 | 46/18888 | 0.044 | 41.06 | 8.8566 | 0.001 | 0.016 | CHMP3/TARDBP |
| GO:0090224 | regulation of spindle organization | 2/20 | 46/18888 | 0.044 | 41.06 | 8.8566 | 0.001 | 0.016 | CHMP3/CLTC |
| GO:1905144 | response to acetylcholine | 2/20 | 46/18888 | 0.044 | 41.06 | 8.8566 | 0.001 | 0.016 | ITPR1/PLCB1 |
| GO:0008154 | actin polymerization or depolymerization | 3/20 | 196/18888 | 0.015 | 14.46 | 6.1648 | 0.001 | 0.016 | WASL/RAC1/RICTOR |
| GO:0051650 | establishment of vesicle localization | 3/20 | 196/18888 | 0.015 | 14.46 | 6.1648 | 0.001 | 0.016 | WASL/HGS/STAM |
| GO:0090276 | regulation of peptide hormone secretion | 3/20 | 197/18888 | 0.015 | 14.38 | 6.1470 | 0.001 | 0.016 | TARDBP/ITPR1/PLCB1 |
| GO:0006623 | protein targeting to vacuole | 2/20 | 48/18888 | 0.042 | 39.35 | 8.6612 | 0.001 | 0.016 | HGS/HSPA8 |
| GO:0072583 | clathrin-dependent endocytosis | 2/20 | 48/18888 | 0.042 | 39.35 | 8.6612 | 0.001 | 0.016 | WASL/CLTC |
| GO:0002791 | regulation of peptide secretion | 3/20 | 200/18888 | 0.015 | 14.17 | 6.0943 | 0.001 | 0.016 | TARDBP/ITPR1/PLCB1 |
| GO:0030073 | insulin secretion | 3/20 | 200/18888 | 0.015 | 14.17 | 6.0943 | 0.001 | 0.016 | TARDBP/ITPR1/PLCB1 |
| GO:0072594 | establishment of protein localization to organelle | 4/20 | 454/18888 | 0.009 | 8.32 | 5.1405 | 0.001 | 0.016 | TARDBP/HGS/STAM/HSPA8 |
| GO:0090087 | regulation of peptide transport | 3/20 | 202/18888 | 0.015 | 14.03 | 6.0597 | 0.001 | 0.016 | TARDBP/ITPR1/PLCB1 |
| GO:0098926 | postsynaptic signal transduction | 2/20 | 49/18888 | 0.041 | 38.55 | 8.5680 | 0.001 | 0.016 | ITPR1/PLCB1 |
| GO:0098927 | vesicle-mediated transport between endosomal compartments | 2/20 | 49/18888 | 0.041 | 38.55 | 8.5680 | 0.001 | 0.016 | CHMP3/MAPK1 |
| GO:0071674 | mononuclear cell migration | 3/20 | 206/18888 | 0.015 | 13.75 | 5.9921 | 0.001 | 0.016 | WASL/PLCB1/MAPK1 |
| GO:0071622 | regulation of granulocyte chemotaxis | 2/20 | 51/18888 | 0.039 | 37.04 | 8.3896 | 0.001 | 0.017 | RAC1/MAPK1 |
| GO:0071383 | cellular response to steroid hormone stimulus | 3/20 | 213/18888 | 0.014 | 13.30 | 5.8782 | 0.001 | 0.018 | ESR1/MAPK1/HSPA8 |
| GO:0048009 | insulin-like growth factor receptor signaling pathway | 2/20 | 53/18888 | 0.038 | 35.64 | 8.2213 | 0.001 | 0.018 | PLCB1/MAPK1 |
| GO:0051648 | vesicle localization | 3/20 | 215/18888 | 0.014 | 13.18 | 5.8467 | 0.001 | 0.018 | WASL/HGS/STAM |
| GO:0002064 | epithelial cell development | 3/20 | 216/18888 | 0.014 | 13.12 | 5.8311 | 0.002 | 0.018 | RHEB/PLCB1/ESR1 |
| GO:0002686 | negative regulation of leukocyte migration | 2/20 | 55/18888 | 0.036 | 34.34 | 8.0620 | 0.002 | 0.018 | WASL/PLCB1 |
| GO:0006998 | nuclear envelope organization | 2/20 | 55/18888 | 0.036 | 34.34 | 8.0620 | 0.002 | 0.018 | CHMP3/TARDBP |
| GO:0031667 | response to nutrient levels | 4/20 | 490/18888 | 0.008 | 7.71 | 4.8992 | 0.002 | 0.019 | RHEB/MAPK1/HSPA8/RICTOR |
| GO:0051651 | maintenance of location in cell | 3/20 | 228/18888 | 0.013 | 12.43 | 5.6513 | 0.002 | 0.02 | FKBP1A/ITPR1/PLCB1 |
| GO:0046173 | polyol biosynthetic process | 2/20 | 60/18888 | 0.033 | 31.48 | 7.6988 | 0.002 | 0.021 | IPMK/ITPKB |
| GO:0061462 | protein localization to lysosome | 2/20 | 60/18888 | 0.033 | 31.48 | 7.6988 | 0.002 | 0.021 | HGS/HSPA8 |
| GO:0097529 | myeloid leukocyte migration | 3/20 | 239/18888 | 0.013 | 11.85 | 5.4981 | 0.002 | 0.022 | PLCB1/RAC1/MAPK1 |
| GO:0030072 | peptide hormone secretion | 3/20 | 242/18888 | 0.012 | 11.71 | 5.4580 | 0.002 | 0.023 | TARDBP/ITPR1/PLCB1 |
| GO:0010324 | membrane invagination | 2/20 | 65/18888 | 0.031 | 29.06 | 7.3775 | 0.002 | 0.024 | HGS/RAC1 |
| GO:0002790 | peptide secretion | 3/20 | 247/18888 | 0.012 | 11.47 | 5.3928 | 0.002 | 0.024 | TARDBP/ITPR1/PLCB1 |
| GO:0051222 | positive regulation of protein transport | 3/20 | 251/18888 | 0.012 | 11.29 | 5.3419 | 0.002 | 0.024 | TARDBP/ITPR1/PLCB1 |
| GO:0061180 | mammary gland epithelium development | 2/20 | 67/18888 | 0.03 | 28.19 | 7.2590 | 0.002 | 0.024 | ESR1/MAPK1 |
| GO:0032984 | protein-containing complex disassembly | 3/20 | 252/18888 | 0.012 | 11.24 | 5.3294 | 0.002 | 0.024 | CHMP3/CLTC/HSPA8 |
| GO:0046883 | regulation of hormone secretion | 3/20 | 254/18888 | 0.012 | 11.15 | 5.3046 | 0.002 | 0.025 | TARDBP/ITPR1/PLCB1 |
| GO:0032886 | regulation of microtubule-based process | 3/20 | 258/18888 | 0.012 | 10.98 | 5.2557 | 0.002 | 0.026 | CHMP3/CLTC/RAC1 |
| GO:0032008 | positive regulation of TOR signaling | 2/20 | 72/18888 | 0.028 | 26.23 | 6.9841 | 0.003 | 0.027 | RHEB/RICTOR |
| GO:0032386 | regulation of intracellular transport | 3/20 | 267/18888 | 0.011 | 10.61 | 5.1495 | 0.003 | 0.028 | CHMP3/TARDBP/MAPK1 |
| GO:0050708 | regulation of protein secretion | 3/20 | 269/18888 | 0.011 | 10.53 | 5.1266 | 0.003 | 0.028 | TARDBP/ITPR1/PLCB1 |
| GO:0015833 | peptide transport | 3/20 | 271/18888 | 0.011 | 10.45 | 5.1040 | 0.003 | 0.028 | TARDBP/ITPR1/PLCB1 |
| GO:0051057 | positive regulation of small GTPase mediated signal transduction | 2/20 | 76/18888 | 0.026 | 24.85 | 6.7836 | 0.003 | 0.029 | RAC1/ITPKB |
| GO:0045921 | positive regulation of exocytosis | 2/20 | 77/18888 | 0.026 | 24.53 | 6.7359 | 0.003 | 0.03 | HGS/STAM |
| GO:0006874 | intracellular calcium ion homeostasis | 3/20 | 302/18888 | 0.01 | 9.38 | 4.7804 | 0.004 | 0.037 | FKBP1A/ITPR1/PLCB1 |
| GO:0046879 | hormone secretion | 3/20 | 311/18888 | 0.01 | 9.11 | 4.6951 | 0.004 | 0.04 | TARDBP/ITPR1/PLCB1 |
| GO:0032273 | positive regulation of protein polymerization | 2/20 | 91/18888 | 0.022 | 20.76 | 6.1505 | 0.004 | 0.04 | RAC1/RICTOR |
| GO:0042886 | amide transport | 3/20 | 312/18888 | 0.01 | 9.08 | 4.6858 | 0.004 | 0.04 | TARDBP/ITPR1/PLCB1 |
| GO:0002690 | positive regulation of leukocyte chemotaxis | 2/20 | 93/18888 | 0.022 | 20.31 | 6.0776 | 0.004 | 0.041 | RAC1/MAPK1 |
| GO:1903432 | regulation of TORC1 signaling | 2/20 | 93/18888 | 0.022 | 20.31 | 6.0776 | 0.004 | 0.041 | RHEB/AKT1S1 |
| GO:0009914 | hormone transport | 3/20 | 322/18888 | 0.009 | 8.80 | 4.5954 | 0.005 | 0.042 | TARDBP/ITPR1/PLCB1 |
| GO:0031647 | regulation of protein stability | 3/20 | 326/18888 | 0.009 | 8.69 | 4.5604 | 0.005 | 0.043 | TARDBP/MAPK1/HSPA8 |
| GO:0055074 | calcium ion homeostasis | 3/20 | 326/18888 | 0.009 | 8.69 | 4.5604 | 0.005 | 0.043 | FKBP1A/ITPR1/PLCB1 |
| GO:1904951 | positive regulation of establishment of protein localization | 3/20 | 326/18888 | 0.009 | 8.69 | 4.5604 | 0.005 | 0.043 | TARDBP/ITPR1/PLCB1 |
| GO:0038202 | TORC1 signaling | 2/20 | 99/18888 | 0.02 | 19.08 | 5.8718 | 0.005 | 0.044 | RHEB/AKT1S1 |
| GO:0070588 | calcium ion transmembrane transport | 3/20 | 334/18888 | 0.009 | 8.48 | 4.4920 | 0.005 | 0.045 | FKBP1A/ITPR1/PLCB1 |
| GO:0051235 | maintenance of location | 3/20 | 337/18888 | 0.009 | 8.41 | 4.4670 | 0.005 | 0.046 | FKBP1A/ITPR1/PLCB1 |
| GO:0008585 | female gonad development | 2/20 | 102/18888 | 0.02 | 18.52 | 5.7755 | 0.005 | 0.046 | ESR1/UBB |
| GO:0048545 | response to steroid hormone | 3/20 | 338/18888 | 0.009 | 8.38 | 4.4587 | 0.005 | 0.046 | ESR1/MAPK1/HSPA8 |
| GO:0046545 | development of primary female sexual characteristics | 2/20 | 106/18888 | 0.019 | 17.82 | 5.6534 | 0.006 | 0.049 | ESR1/UBB |
| **Cellular components (CC)** | | | | | | | | | |
| GO:0036452 | ESCRT complex | 3/20 | 29/19894 | 0.103 | 102.90 | 17.4201 | <0.001 | 0.001 | CHMP3/HGS/STAM |
| GO:0038201 | TOR complex | 2/20 | 12/19894 | 0.167 | 165.78 | 18.1132 | <0.001 | 0.005 | AKT1S1/RICTOR |
| GO:0042470 | melanosome | 3/20 | 112/19894 | 0.027 | 26.64 | 8.6333 | <0.001 | 0.007 | CLTC/RAC1/HSPA8 |
| GO:0048770 | pigment granule | 3/20 | 112/19894 | 0.027 | 26.64 | 8.6333 | <0.001 | 0.007 | CLTC/RAC1/HSPA8 |
| GO:0030139 | endocytic vesicle | 4/20 | 350/19894 | 0.011 | 11.37 | 6.2079 | <0.001 | 0.009 | WASL/CLTC/HGS/UBB |
| GO:0032585 | multivesicular body membrane | 2/20 | 29/19894 | 0.069 | 68.60 | 11.5564 | <0.001 | 0.009 | CHMP3/HGS |
| GO:0005925 | focal adhesion | 4/20 | 421/19894 | 0.01 | 9.45 | 5.5596 | 0.001 | 0.011 | CLTC/RAC1/MAPK1/HSPA8 |
| GO:0005769 | early endosome | 4/20 | 428/19894 | 0.009 | 9.30 | 5.5041 | 0.001 | 0.011 | CHMP3/HGS/STAM/MAPK1 |
| GO:0030055 | cell-substrate junction | 4/20 | 431/19894 | 0.009 | 9.23 | 5.4807 | 0.001 | 0.011 | CLTC/RAC1/MAPK1/HSPA8 |
| GO:0101002 | ficolin-1-rich granule | 3/20 | 185/19894 | 0.016 | 16.13 | 6.5588 | 0.001 | 0.011 | RAC1/MAPK1/HSPA8 |
| GO:0005765 | lysosomal membrane | 4/20 | 443/19894 | 0.009 | 8.98 | 5.3894 | 0.001 | 0.011 | CHMP3/RHEB/CLTC/HSPA8 |
| GO:0098852 | lytic vacuole membrane | 4/20 | 443/19894 | 0.009 | 8.98 | 5.3894 | 0.001 | 0.011 | CHMP3/RHEB/CLTC/HSPA8 |
| GO:0030666 | endocytic vesicle membrane | 3/20 | 204/19894 | 0.015 | 14.63 | 6.2065 | 0.001 | 0.012 | WASL/CLTC/UBB |
| GO:0005774 | vacuolar membrane | 4/20 | 487/19894 | 0.008 | 8.17 | 5.0819 | 0.001 | 0.013 | CHMP3/RHEB/CLTC/HSPA8 |
| GO:0005771 | multivesicular body | 2/20 | 68/19894 | 0.029 | 29.26 | 7.4040 | 0.002 | 0.02 | CHMP3/HGS |
| GO:0016529 | sarcoplasmic reticulum | 2/20 | 76/19894 | 0.026 | 26.18 | 6.9758 | 0.003 | 0.024 | FKBP1A/ITPR1 |
| GO:0005876 | spindle microtubule | 2/20 | 82/19894 | 0.024 | 24.26 | 6.6957 | 0.003 | 0.026 | CHMP3/CLTC |
| GO:0005770 | late endosome | 3/20 | 314/19894 | 0.01 | 9.50 | 4.8181 | 0.004 | 0.029 | CHMP3/HGS/MAPK1 |
| GO:0016528 | sarcoplasm | 2/20 | 95/19894 | 0.021 | 20.94 | 6.1803 | 0.004 | 0.031 | FKBP1A/ITPR1 |
| GO:1904813 | ficolin-1-rich granule lumen | 2/20 | 124/19894 | 0.016 | 16.04 | 5.3307 | 0.007 | 0.049 | MAPK1/HSPA8 |
| **Molecular functions (MF)** | | | | | | | | | |
| GO:0035091 | phosphatidylinositol binding | 5/20 | 276/18522 | 0.018 | 16.78 | 8.6824 | <0.001 | 0.001 | CHMP3/ITPR1/PLCB1/HGS/STAM |
| GO:0180030 | GO:0180030 | 2/20 | 11/18522 | 0.182 | 168.38 | 18.2569 | <0.001 | 0.004 | IPMK/ITPKB |
| GO:0005543 | phospholipid binding | 5/20 | 487/18522 | 0.01 | 9.51 | 6.2558 | <0.001 | 0.007 | CHMP3/ITPR1/PLCB1/HGS/STAM |
| GO:1990381 | ubiquitin-specific protease binding | 2/20 | 21/18522 | 0.095 | 88.20 | 13.1452 | <0.001 | 0.008 | CHMP3/CLTC |
| GO:0044389 | ubiquitin-like protein ligase binding | 4/20 | 324/18522 | 0.012 | 11.43 | 6.2290 | <0.001 | 0.01 | HGS/UBB/STAM/HSPA8 |
| GO:0005516 | calmodulin binding | 3/20 | 205/18522 | 0.015 | 13.55 | 5.9419 | 0.001 | 0.032 | PLCB1/ESR1/ITPKB |
| GO:0019887 | protein kinase regulator activity | 3/20 | 232/18522 | 0.013 | 11.98 | 5.5309 | 0.002 | 0.039 | RHEB/AKT1S1/RICTOR |
| GO:0043539 | protein serine/threonine kinase activator activity | 2/20 | 65/18522 | 0.031 | 28.50 | 7.3009 | 0.002 | 0.04 | RHEB/RICTOR |
| GO:0019207 | kinase regulator activity | 3/20 | 270/18522 | 0.011 | 10.29 | 5.0557 | 0.003 | 0.047 | RHEB/AKT1S1/RICTOR |

**Table S8** Significantly enriched Kyoto Encyclopaedia Genes and Genomes (KEGG) terms of top 20 hub genes identified from PPI network. A total of 74 significantly enriched KEGG pathways were identified in this study.

| **Category** | **Subcategory** | **ID** | **Description** | **Gene Ratio** | **Bg Ratio** | **Rich Factor** | **Fold Enrichment** | **zScore** | ***p* value** | ***p* adjust** |
| --- | --- | --- | --- | --- | --- | --- | --- | --- | --- | --- |
| Human Diseases | Infectious disease: bacterial | hsa05131 | Shigellosis | 7/19 | 247/8659 | 0.028 | 12.92 | 8.9093 | <0.001 | <0.001 |
| Environmental Information Processing | Signal transduction | hsa04070 | Phosphatidylinositol signaling system | 5/19 | 97/8659 | 0.052 | 23.49 | 10.4459 | <0.001 | <0.001 |
| Organismal Systems | Endocrine system | hsa04915 | Estrogen signaling pathway | 5/19 | 137/8659 | 0.037 | 16.63 | 8.6487 | <0.001 | 0.001 |
| Cellular Processes | Transport and catabolism | hsa04144 | Endocytosis | 6/19 | 250/8659 | 0.024 | 10.94 | 7.4767 | <0.001 | 0.001 |
| Metabolism | Carbohydrate metabolism | hsa00562 | Inositol phosphate metabolism | 4/19 | 73/8659 | 0.055 | 24.97 | 9.6449 | <0.001 | 0.001 |
| Cellular Processes | Transport and catabolism | hsa04140 | Autophagy - animal | 5/19 | 165/8659 | 0.03 | 13.81 | 7.7906 | <0.001 | 0.001 |
| Human Diseases | Cardiovascular disease | hsa05417 | Lipid and atherosclerosis | 5/19 | 215/8659 | 0.023 | 10.60 | 6.6831 | <0.001 | 0.002 |
| Human Diseases | Infectious disease: viral | hsa05163 | Human cytomegalovirus infection | 5/19 | 225/8659 | 0.022 | 10.13 | 6.5051 | <0.001 | 0.002 |
| Organismal Systems | Endocrine system | hsa04919 | Thyroid hormone signaling pathway | 4/19 | 121/8659 | 0.033 | 15.07 | 7.3064 | <0.001 | 0.002 |
| Organismal Systems | Excretory system | hsa04961 | Endocrine and other factor-regulated calcium reabsorption | 3/19 | 53/8659 | 0.057 | 25.80 | 8.4909 | <0.001 | 0.003 |
| Organismal Systems | Nervous system | hsa04730 | Long-term depression | 3/19 | 60/8659 | 0.05 | 22.79 | 7.9410 | <0.001 | 0.004 |
| Environmental Information Processing | Signal transduction | hsa04150 | mTOR signaling pathway | 4/19 | 156/8659 | 0.026 | 11.69 | 6.3154 | <0.001 | 0.004 |
| Organismal Systems | Endocrine system | hsa04929 | GnRH secretion | 3/19 | 64/8659 | 0.047 | 21.36 | 7.6671 | <0.001 | 0.004 |
| Human Diseases | Neurodegenerative disease | hsa05022 | Pathways of neurodegeneration - multiple diseases | 6/19 | 476/8659 | 0.013 | 5.74 | 4.9931 | <0.001 | 0.004 |
| Organismal Systems | Nervous system | hsa04720 | Long-term potentiation | 3/19 | 67/8659 | 0.045 | 20.41 | 7.4775 | <0.001 | 0.004 |
| Human Diseases | Infectious disease: bacterial | hsa05100 | Bacterial invasion of epithelial cells | 3/19 | 77/8659 | 0.039 | 17.76 | 6.9255 | 0.001 | 0.006 |
| Human Diseases | Infectious disease: viral | hsa05167 | Kaposi sarcoma-associated herpesvirus infection | 4/19 | 194/8659 | 0.021 | 9.40 | 5.5465 | 0.001 | 0.007 |
| Cellular Processes | Cellular community - eukaryotes | hsa04540 | Gap junction | 3/19 | 88/8659 | 0.034 | 15.54 | 6.4271 | 0.001 | 0.008 |
| Human Diseases | Cancer: overview | hsa05205 | Proteoglycans in cancer | 4/19 | 205/8659 | 0.02 | 8.89 | 5.3627 | 0.001 | 0.008 |
| Cellular Processes | Cellular community - eukaryotes | hsa04520 | Adherens junction | 3/19 | 93/8659 | 0.032 | 14.70 | 6.2293 | 0.001 | 0.008 |
| Organismal Systems | Endocrine system | hsa04912 | GnRH signaling pathway | 3/19 | 93/8659 | 0.032 | 14.70 | 6.2293 | 0.001 | 0.008 |
| Organismal Systems | Environmental adaptation | hsa04713 | Circadian entrainment | 3/19 | 97/8659 | 0.031 | 14.09 | 6.0818 | 0.001 | 0.009 |
| Human Diseases | Cancer: overview | hsa05231 | Choline metabolism in cancer | 3/19 | 98/8659 | 0.031 | 13.95 | 6.0463 | 0.001 | 0.009 |
| Human Diseases | Endocrine and metabolic disease | hsa04933 | AGE-RAGE signaling pathway in diabetic complications | 3/19 | 100/8659 | 0.03 | 13.67 | 5.9768 | 0.001 | 0.009 |
| Organismal Systems | Digestive system | hsa04972 | Pancreatic secretion | 3/19 | 102/8659 | 0.029 | 13.40 | 5.9092 | 0.001 | 0.009 |
| Organismal Systems | Endocrine system | hsa04928 | Parathyroid hormone synthesis, secretion and action | 3/19 | 106/8659 | 0.028 | 12.90 | 5.7797 | 0.002 | 0.01 |
| Organismal Systems | Nervous system | hsa04725 | Cholinergic synapse | 3/19 | 113/8659 | 0.027 | 12.10 | 5.5690 | 0.002 | 0.011 |
| Organismal Systems | Nervous system | hsa04724 | Glutamatergic synapse | 3/19 | 115/8659 | 0.026 | 11.89 | 5.5122 | 0.002 | 0.011 |
| Organismal Systems | Nervous system | hsa04726 | Serotonergic synapse | 3/19 | 115/8659 | 0.026 | 11.89 | 5.5122 | 0.002 | 0.011 |
| Organismal Systems | Endocrine system | hsa04935 | Growth hormone synthesis, secretion and action | 3/19 | 120/8659 | 0.025 | 11.39 | 5.3762 | 0.002 | 0.012 |
| Environmental Information Processing | Signal transduction | hsa04071 | Sphingolipid signaling pathway | 3/19 | 121/8659 | 0.025 | 11.30 | 5.3499 | 0.002 | 0.012 |
| Organismal Systems | Immune system | hsa04611 | Platelet activation | 3/19 | 124/8659 | 0.024 | 11.03 | 5.2730 | 0.002 | 0.012 |
| Human Diseases | Neurodegenerative disease | hsa05020 | Prion disease | 4/19 | 272/8659 | 0.015 | 6.70 | 4.4806 | 0.003 | 0.013 |
| Organismal Systems | Circulatory system | hsa04270 | Vascular smooth muscle contraction | 3/19 | 134/8659 | 0.022 | 10.20 | 5.0346 | 0.003 | 0.014 |
| Organismal Systems | Development and regeneration | hsa04380 | Osteoclast differentiation | 3/19 | 135/8659 | 0.022 | 10.13 | 5.0122 | 0.003 | 0.014 |
| Organismal Systems | Endocrine system | hsa04910 | Insulin signaling pathway | 3/19 | 137/8659 | 0.022 | 9.98 | 4.9679 | 0.003 | 0.014 |
| Human Diseases | Infectious disease: bacterial | hsa05135 | Yersinia infection | 3/19 | 137/8659 | 0.022 | 9.98 | 4.9679 | 0.003 | 0.014 |
| Environmental Information Processing | Signal transduction | hsa04371 | Apelin signaling pathway | 3/19 | 139/8659 | 0.022 | 9.84 | 4.9246 | 0.003 | 0.014 |
| Environmental Information Processing | Signal transduction | hsa04072 | Phospholipase D signaling pathway | 3/19 | 148/8659 | 0.02 | 9.24 | 4.7401 | 0.004 | 0.016 |
| Organismal Systems | Nervous system | hsa04723 | Retrograde endocannabinoid signaling | 3/19 | 148/8659 | 0.02 | 9.24 | 4.7401 | 0.004 | 0.016 |
| Organismal Systems | Endocrine system | hsa04921 | Oxytocin signaling pathway | 3/19 | 154/8659 | 0.02 | 8.88 | 4.6256 | 0.004 | 0.018 |
| Human Diseases | Endocrine and metabolic disease | hsa04934 | Cushing syndrome | 3/19 | 155/8659 | 0.019 | 8.82 | 4.6071 | 0.004 | 0.018 |
| Cellular Processes | Cell growth and death | hsa04218 | Cellular senescence | 3/19 | 156/8659 | 0.019 | 8.76 | 4.5888 | 0.005 | 0.018 |
| Environmental Information Processing | Signal transduction | hsa04022 | cGMP-PKG signaling pathway | 3/19 | 167/8659 | 0.018 | 8.19 | 4.3977 | 0.005 | 0.021 |
| Environmental Information Processing | Signal transduction | hsa04370 | VEGF signaling pathway | 2/19 | 59/8659 | 0.034 | 15.45 | 5.2220 | 0.007 | 0.027 |
| Organismal Systems | Immune system | hsa04621 | NOD-like receptor signaling pathway | 3/19 | 186/8659 | 0.016 | 7.35 | 4.1056 | 0.007 | 0.027 |
| Organismal Systems | Aging | hsa04213 | Longevity regulating pathway - multiple species | 2/19 | 61/8659 | 0.033 | 14.94 | 5.1242 | 0.008 | 0.028 |
| Organismal Systems | Immune system | hsa04613 | Neutrophil extracellular trap formation | 3/19 | 191/8659 | 0.016 | 7.16 | 4.0356 | 0.008 | 0.028 |
| Organismal Systems | Immune system | hsa04062 | Chemokine signaling pathway | 3/19 | 192/8659 | 0.016 | 7.12 | 4.0219 | 0.008 | 0.028 |
| Human Diseases | Infectious disease: bacterial | hsa05130 | Pathogenic Escherichia coli infection | 3/19 | 198/8659 | 0.015 | 6.91 | 3.9416 | 0.009 | 0.029 |
| Organismal Systems | Endocrine system | hsa04927 | Cortisol synthesis and secretion | 2/19 | 65/8659 | 0.031 | 14.02 | 4.9418 | 0.009 | 0.029 |
| Organismal Systems | Immune system | hsa04664 | Fc epsilon RI signaling pathway | 2/19 | 68/8659 | 0.029 | 13.40 | 4.8153 | 0.01 | 0.031 |
| Organismal Systems | Endocrine system | hsa04924 | Renin secretion | 2/19 | 69/8659 | 0.029 | 13.21 | 4.7749 | 0.01 | 0.031 |
| Human Diseases | Cancer: specific types | hsa05211 | Renal cell carcinoma | 2/19 | 69/8659 | 0.029 | 13.21 | 4.7749 | 0.01 | 0.031 |
| Organismal Systems | Endocrine system | hsa04917 | Prolactin signaling pathway | 2/19 | 70/8659 | 0.029 | 13.02 | 4.7353 | 0.01 | 0.031 |
| Environmental Information Processing | Signal transduction | hsa04015 | Rap1 signaling pathway | 3/19 | 210/8659 | 0.014 | 6.51 | 3.7908 | 0.01 | 0.031 |
| Human Diseases | Infectious disease: viral | hsa05170 | Human immunodeficiency virus 1 infection | 3/19 | 212/8659 | 0.014 | 6.45 | 3.7668 | 0.011 | 0.031 |
| Organismal Systems | Endocrine system | hsa04918 | Thyroid hormone synthesis | 2/19 | 75/8659 | 0.027 | 12.15 | 4.5489 | 0.012 | 0.033 |
| Organismal Systems | Digestive system | hsa04971 | Gastric acid secretion | 2/19 | 76/8659 | 0.026 | 11.99 | 4.5137 | 0.012 | 0.033 |
| Human Diseases | Cancer: specific types | hsa05212 | Pancreatic cancer | 2/19 | 76/8659 | 0.026 | 11.99 | 4.5137 | 0.012 | 0.033 |
| Cellular Processes | Cell motility | hsa04810 | Regulation of actin cytoskeleton | 3/19 | 229/8659 | 0.013 | 5.97 | 3.5745 | 0.013 | 0.036 |
| Organismal Systems | Immune system | hsa04662 | B cell receptor signaling pathway | 2/19 | 84/8659 | 0.024 | 10.85 | 4.2543 | 0.014 | 0.039 |
| Human Diseases | Cancer: specific types | hsa05210 | Colorectal cancer | 2/19 | 86/8659 | 0.023 | 10.60 | 4.1948 | 0.015 | 0.04 |
| Organismal Systems | Aging | hsa04211 | Longevity regulating pathway | 2/19 | 89/8659 | 0.023 | 10.24 | 4.1093 | 0.016 | 0.042 |
| Human Diseases | Infectious disease: bacterial | hsa05132 | Salmonella infection | 3/19 | 249/8659 | 0.012 | 5.49 | 3.3717 | 0.016 | 0.042 |
| Environmental Information Processing | Signal transduction | hsa04020 | Calcium signaling pathway | 3/19 | 253/8659 | 0.012 | 5.40 | 3.3338 | 0.017 | 0.043 |
| Organismal Systems | Digestive system | hsa04970 | Salivary secretion | 2/19 | 93/8659 | 0.022 | 9.80 | 4.0013 | 0.017 | 0.044 |
| Organismal Systems | Immune system | hsa04666 | Fc gamma R-mediated phagocytosis | 2/19 | 97/8659 | 0.021 | 9.40 | 3.8997 | 0.019 | 0.046 |
| Human Diseases | Drug resistance: antineoplastic | hsa01522 | Endocrine resistance | 2/19 | 98/8659 | 0.02 | 9.30 | 3.8752 | 0.019 | 0.046 |
| Organismal Systems | Sensory system | hsa04750 | Inflammatory mediator regulation of TRP channels | 2/19 | 98/8659 | 0.02 | 9.30 | 3.8752 | 0.019 | 0.046 |
| Organismal Systems | Endocrine system | hsa04925 | Aldosterone synthesis and secretion | 2/19 | 98/8659 | 0.02 | 9.30 | 3.8752 | 0.019 | 0.046 |
| Organismal Systems | Endocrine system | hsa04916 | Melanogenesis | 2/19 | 101/8659 | 0.02 | 9.02 | 3.8038 | 0.02 | 0.048 |
| Human Diseases | Infectious disease: parasitic | hsa05142 | Chagas disease | 2/19 | 102/8659 | 0.02 | 8.94 | 3.7807 | 0.021 | 0.048 |
| Organismal Systems | Immune system | hsa04625 | C-type lectin receptor signaling pathway | 2/19 | 104/8659 | 0.019 | 8.76 | 3.7353 | 0.021 | 0.049 |
